# Acetylated KIAA1429 by TIP60 facilitates metastasis and immune evasion of hepatocellular carcinoma via N6-methyladenosine-KDM5B-mediated regulation of FoxO1

**DOI:** 10.1038/s41420-025-02462-4

**Published:** 2025-04-29

**Authors:** Hu Quan, Huijun Zhou, Fei Chen, Jie Chen, Yun He, Hua Xiao, Jia Liu, Lei Shi, Wei Xie, Pan Chen, Jia Luo

**Affiliations:** 1 Hunan Cancer Hospital and the Affiliated Cancer Hospital of Xiangya School of Medicine, Central South University, Changsha, 410013 Hunan Province P.R. China; 2 Department of general Surgery, Turpan City People’s Hospital, Tulufan, 838000 Xinjiang P.R. China; 3 The Central Hospital of Shaoyang, The Affiliated Shaoyang Hospital, Hengyang Medical School, University of South China, Hengyang, 421001 Hunan Province P.R. China

**Keywords:** Cancer, Diseases

## Abstract

Hepatocellular carcinoma (HCC) is characterized by programmed cell death ligand-1 (PD-L1)-mediated immune escape. This study aimed to elucidate the function and mechanism behind KIAA1429, a component of N6-methyladenosine (m^6^A) complex, in immune escape of HCC. PD-L1 expression was assessed through immunofluorescence staining, and flow cytometry was used to determine CD8^+^ T cell percentage. The level of IFN-γ was detected using enzyme-linked immunosorbent assay. Cell proliferation, migration, and invasion were evaluated through CCK-8, colony formation, and Transwell assays, respectively. The m^6^A modification level was measured using an RNA methylation quantification assay, m^6^A dot blot, and methylated RNA immunoprecipitation-qPCR. Molecule interaction was validated using RNA pulldown, RNA immunoprecipitation, chromatin immunoprecipitation, and co-immunoprecipitation assays. In vivo HCC growth was evaluated in NOD/SCID mice. We found that TIP60, KIAA1429 and KDM5B were highly expressed in HCC cells, while FoxO1 was poorly expressed. Functionally, TIP60/KIAA1429 silencing inhibited PD-L1-mediated HCC immune evasion, growth, migration, and invasion. Mechanistically, TIP60 led to acetylation of KIAA1429, which promoted KDM5B expression in an m^6^A-YTHDF1-dependent manner, and subsequently restrained the transcription and expression of FoxO1. Enforcing YTHDF1 expression or depleting FoxO1 expression markedly reversed the suppressive effect of shKIAA1429 on HCC immune evasion, growth, migration, and invasion. Overall, these findings suggest that acetylated KIAA1429-mediated m^6^A modification endows HCC cells with immune evasion through regulation of KDM5B/FoxO1 axis, which provide a treatment option for HCC by targeting KIAA1429.

## Introduction

Hepatocellular carcinoma (HCC) is the second principal cause of tumor-related deaths worldwide [1], with a 5-year survival rate of only 18%, despite advances in treatment strategies [2]. Programmed cell death-1 (PD-1) is a negative regulator of immunity that binds to the programmed cell death ligand-1 (PD-L1) for transduction of inhibitory signals to T cells and induction of immune escape [3, 4]. HCC cells often overexpress PD-L1, allowing them to evade immune surveillance, and causing immune tolerance and immune evasion [5]. Although PD-L1 inhibition has successfully treated other tumors, its efficacy in HCC has been limited because of immune escape [6]. The molecular mechanisms of HCC immune evasion are poorly understood and uncovering them is crucial in developing effective treatments for HCC.

N6-methyladenosine (m^6^A) is an important RNA modification that modulates RNA degradation, transport, and translation and has a close correlation with the malignant phenotypes of HCC [7]. As a dynamic process, m^6^A modification can be regulated by methyltransferases, demethylases, and effector proteins [8]. m^6^A modification participates in the pathogenesis of various cancers, such as triple negative breast cancer [9], gastric cancer [10], HCC [11], and so on. KIAA1429, also known as vir-Like m^6^A methyltransferase-associated (VIRMA) protein, is a component of an m^6^A complex that has been reported to facilitate HCC development through an m^6^A-methylation mechanism [12]. However, the exact function of KIAA1429 in immune evasion and the detailed modulatory mechanisms in HCC remain largely unknown.

HIV tat interacting protein, 60 kDa (TIP60), also called KAT5, belongs to the MYST family of histone acetyltransferases. TIP60 has been verified to modulate gene expression via acetylation of histones H2A and H4 [13]. TIP60 takes part in diverse pathophysiological processes, including HCC [14]. A previous study documented that TIP60 recruited by circRHOT1 favored HCC development via initiating NR2F6 transcription [15]. So far, whether TIP60-mediated acetylation regulated KIAA1429 expression has not been explored.

Lysine-specific demethylase 5B (KDM5B), is a histone demethylase that is highly expressed in various tumors [16], and is considered as an oncogene in HCC as shown its effects on the promotion of the growth and self-renewal of HCC cells [17]. Previous studies have documented that m6A modification of KDM5B participated in various biological processes. For instance, FTO-mediated m6A modification of KDM5B promoted drug resistance in pancreatic ductal adenocarcinoma via reprogramming lipid accumulation [18]. However, whether KDM5B can be regulated by KIAA1429-mediated m6A modification in HCC has not been clarified. Forkhead box O1 (FoxO1) is recognized as a tumor suppressor that represses growth, metastasis, and chemotherapy resistance in multiple malignancies, including HCC [19–21]. Intriguingly, AnimalTFDB4.0 analysis (http://bioinfo.life.hust.edu.cn/AnimalTFDB4/#/) revealed that KDM5B could bind to the FoxO1 promoter. Thus, we speculated that KDM5B might contribute to HCC progression by suppressing FoxO1 transcription.

Herein, we demonstrated that TIP60-mediated acetylation of KIAA1429 stabilized KDM5B in an m^6^A-dependent manner and subsequently caused transcriptional inhibition of FoxO1, thereby promoting immune evasion of HCC cells. Our findings highlight targeting TIP60/KIAA1429/KDM5B axis as a therapeutic strategy for HCC.

## Results

### High expression of TIP60 conferred immune evasion of HCC cells

**Fig. 1:**
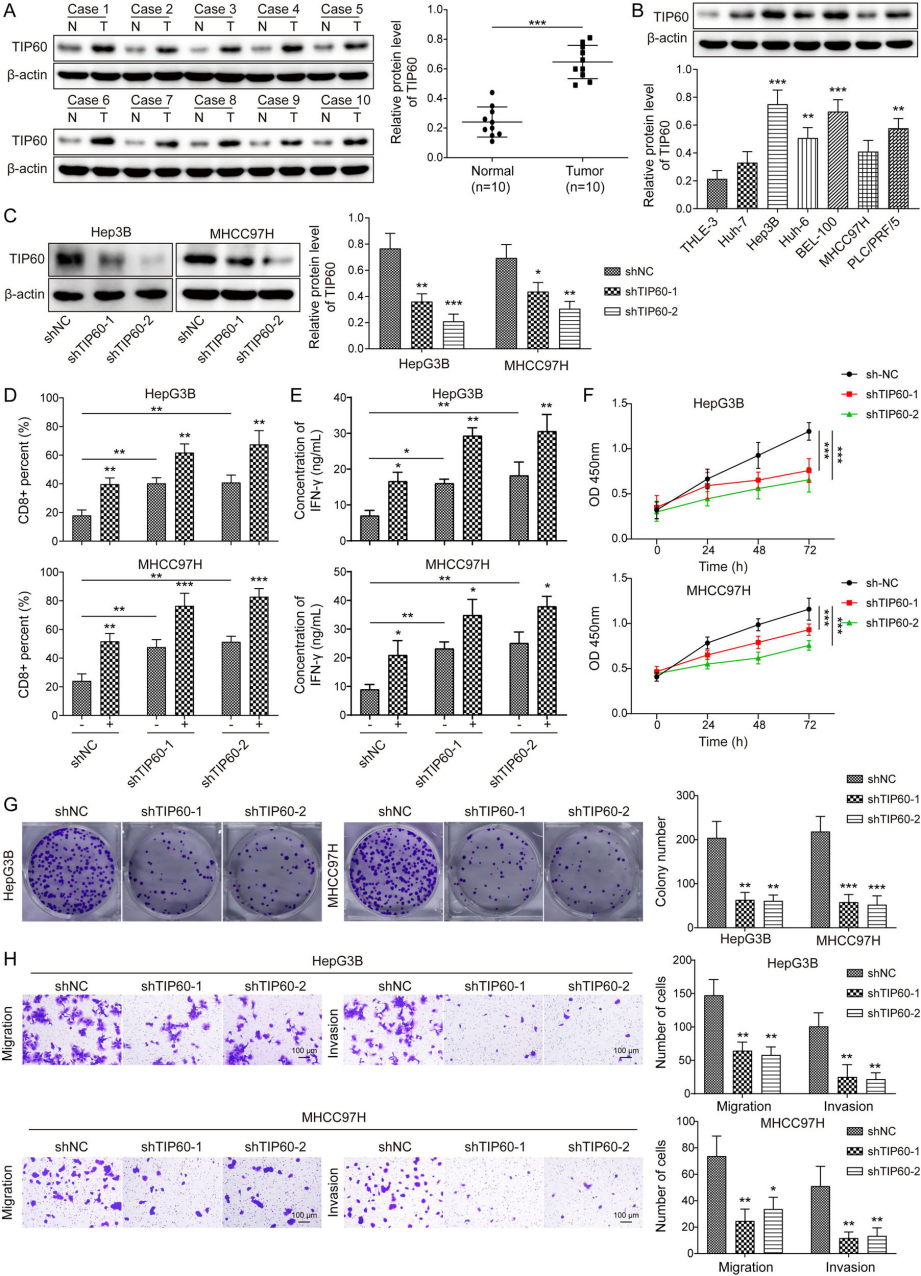
Up-regulation of TIP60 contributed to immune evasion of HCC cells. **A** Western blotting analysis of TIP60 expression in HCC specimens and paired para-tumor tissues (*N* = 10). **B** TIP60 level in multiple HCC cell lines and normal THLE-3 cells was evaluated by western blotting. **C** Hep3B and MHCC97H cells were transfected with shNC or shTIP60-1/-2. Western blotting analysis of TIP60 expression after transfection for 48 h. **D** Flow cytometry assessed the percentage of CD8^+^ T cells in PBMCs co-cultured with HCC cells transfected with shNC or shTIP60-1/-2. **E** ELISA analyzed IFN-γ level in the co-culture of HCC cells transfected with shNC or shTIP60-1/-2 and PBMCs. **F** CCK-8 and (**G**) colony formation assay evaluated the growth of HCC cells transfected with shNC or shTIP60-1/-2. **H** Transwell assay measured the invasion and migration of HCC cells transfected with shNC or shTIP60-1/-2 (Scale bar = 100 μm). All experiments were repeated at least 3 times. Data are presented as the mean ± SD. Statistical significance was determined by Student’s *t* test (for A) or one-way ANOVA (for B-H). *p < 0.05, **p < 0.01, ***p < 0.001.

TIP60 has been revealed to be up-regulated by O-GlcNAcylation, which facilitated HCC metastasis [14]. However, the influence of TIP60 on immune evasion of HCC cells remains ambiguous. To validate differential expression of TIP60 in HCC clinical samples and cells, western blotting was performed. We found that TIP60 protein level was higher in HCC specimens as compared with adjacent normal tissues (Fig. 1A). Consistently, TIP60 protein level was up-regulated in HCC cells in caparison with normal THLE-3 cells (Fig. 1B). Considering that Hep3B and MHCC97H cells possess higher TIP60 level, we selected these two cell lines to knock down TIP60 level via transfection with sh-TIP60#1, 2 (Fig. 1C). To mimic the effect of PD-1 on lymphocytes in tumor microenvironment of HCC, a co-culture model of PBMCs and HCC cells was established. The potential effector functions of T lymphocytes can be evaluated by CD8 and IFN-γ levels. In the coculture system of PBMCs and HCC cells, knockdown of TIP60 or treatment with anti-PD-L1 enhanced T lymphocyte activation, as evidenced by increasing CD8^+^ T cell percentage and IFN-γ level, which was enforced by combination of TIP60 knockdown and PD-L1 antibody (Fig. 1D, E). To further investigate the impact of TIP60 on the malignant phenotypes of HCC cells, CCK-8, colony formation, and Transwell assays were conducted. The results proved that TIP60 deficiency slowed down proliferation (Fig. 1F, G) and repressed migration and invasion of HCC cells (Fig. 1H). These findings suggested that TIP60 up-regulation facilitated the malignant growth and metastasis of HCC cells via immune evasion.

### TIP60 facilitated acetylation of KIAA1429 at the K156 site

**Fig. 2:**
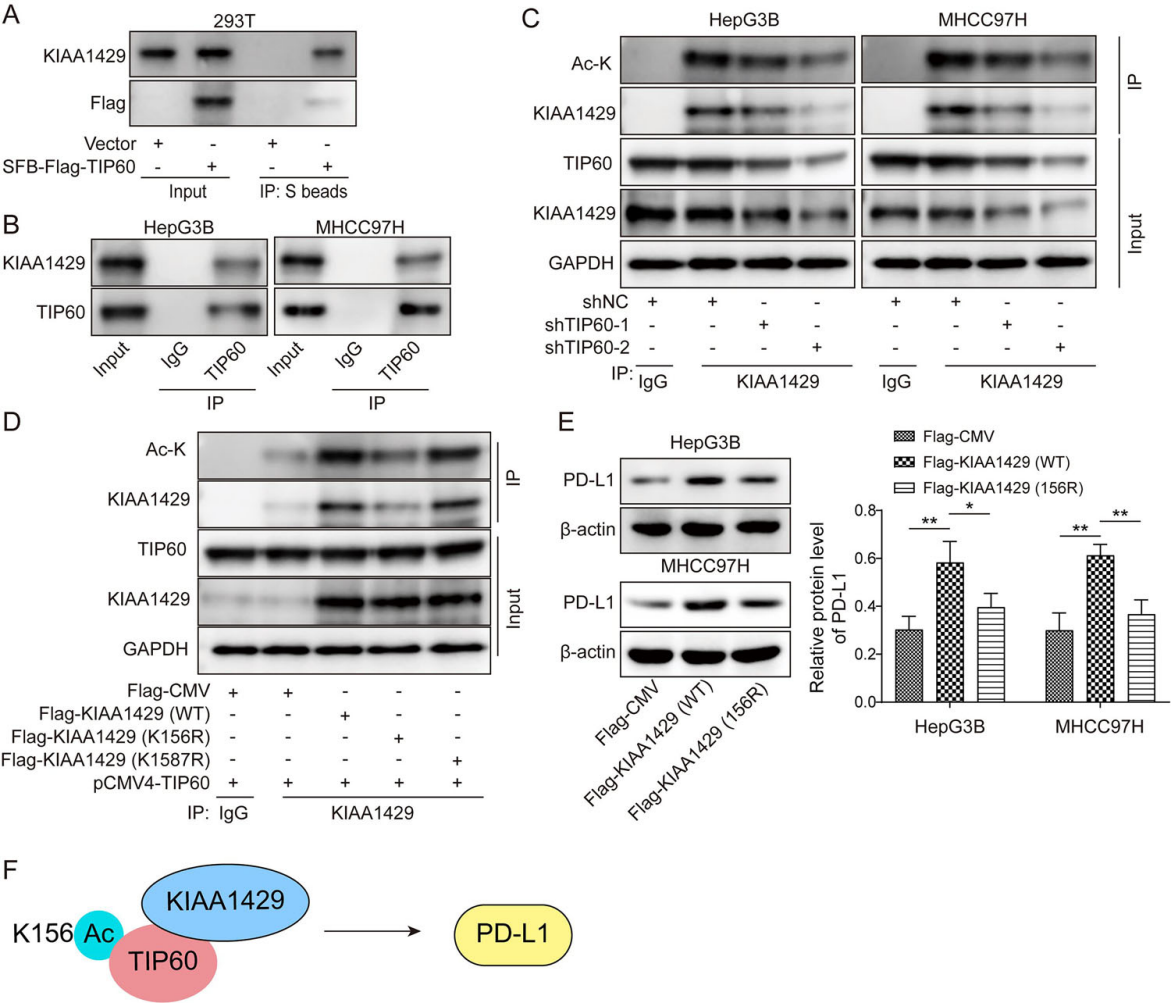
KIAA1429 was acetylated by TIP60 at K156. **A** The exogenous interplay between TIP60 and KIAA1429 proteins was validated by Co-IP. 293 T cells were transfected with vector or SFB-Flag-TIP60, followed by Co-IP and western blotting detection. **B** The endogenous interaction between TIP60 and KIAA1429 proteins was validated by Co-IP. **C** Hep3B and MHCC97H cells were transfected with shNC or shTIP60-1/-2, and the acetylation level of KIAA1429 in HCC cells was detected by Co-IP. **D** 293 T cells were transfected with TIP60 plasmid together with KIAA1429-WT, KIAA1429-K156R, or KIAA1429-K1587R; Co-IP determined the acetylation level of KIAA1429. **E** Western blotting analysis of PD-L1 expression in HCC cells after transfection with Flag-CMV, KIAA1429-WT or KIAA1429-K156R. **F** A flow chart of the results. All experiments were repeated at least 3 times. Data are presented as the mean ± SD. Statistical significance was determined by one-way ANOVA. *p < 0.05, **p < 0.01.

Given that TIP60 is an acetyltransferase that is responsible for acetylation of its target proteins [22], we sought to evaluate whether KIAA1429 could be modulated by TIP60 via acetylation. To investigate the interaction between KIAA1429 and TIP60 in HCC, Co-IP assay was conducted. Co-IP assay validated the exogenous and endogenous interplay between KIAA1429 and TIP60 proteins (Fig. 2A, B). Besides, the localization of KIAA1429 and TIP60 in HCC cells was evaluated by immunofluorescence staining. We found that KIAA1429 with green fluorescence was co-localized with TIP60 with red fluorescence in HCC cells (supplementary Fig. 1A). Similarly, GST pull-down assay confirmed that TIP60 could directly bind to KIAA1429 (supplementary Fig. 1B). In addition, in shTIP60-1/2-transfected HCC cells, the acetylation level of KIAA1429 were declined after immunoprecipitation with KIAA1429 antibody (Fig. 2C). To elucidate the exact acetylation site of KIAA1429, 293 T cells were transfected with KIAA1429-WT, or mutant KIAA1429-K156R/K1587R together with TIP60-overexpression plasmid. Co-IP assay indicated that the acetylation level of KIAA1429 was decreased by transfection with KIAA1429-K156R, but not changed after transfection with KIAA1429-K1587R, suggesting that TIP60 acetylated KIAA1429 at K156 (Fig. 2D). Besides, the protein level of PD-L1 in HCC cells was enhanced by transfection with KIAA1429-WT, rather than KIAA1429-K156R (Fig. 2E). Therefore, TIP60 contributed to KIAA1429 expression via acetylation at K156, which enhanced PD-L1 expression (Fig. 2F).

### KIAA1429 was upregulated and contributed to HCC cell immune evasion

**Fig. 3:**
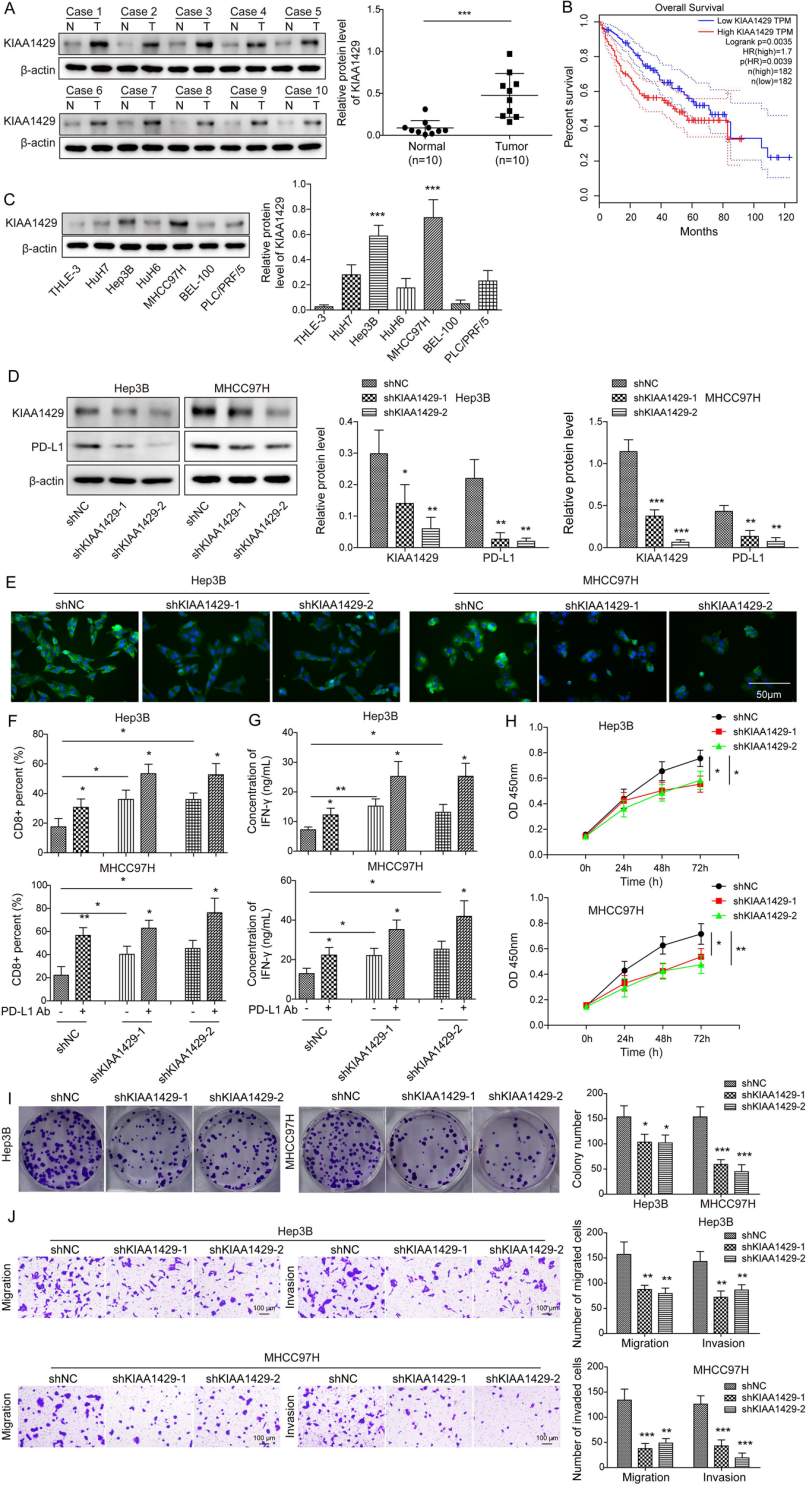
High expression of KIAA1429 led to HCC cell immune evasion. **A** KIAA1429 expression in HCC specimens and paired para-tumor tissues was detected by western blotting (*N* = 10). **B** TCGA database analysis of the correlation between KIAA1429 expression and survival of HCC patients. **C** KIAA1429 levels in various HCC cell lines and normal THLE-3 cells were evaluated by western blotting. **D** Hep3B and MHCC97H cells were transfected with shNC or shKIAA1429-1/-2.Western blotting analysis of KIAA1429 and PD-L1 expression after transfection for 48 h. **E** Immunofluorescence staining of PD-L1 (green) in Hep3B and MHCC97H cells after transfection with shNC or shKIAA1429-1/-2 (Scale bar = 50 µm). **F** Flow cytometry analysis of the proportion of CD8^+^ T cells in PBMCs co-cultured with HCC cells transfected with shNC or shKIAA1429-1/-2. **G** ELISA assessment of IFN-γ level in the co-culture of HCC cells transfected with shNC or shKIAA1429-1/-2 and PBMCs. **H** CCK-8 and (**I**) colony formation assay determined the growth of HCC cells transfected with shNC or shKIAA1429-1/-2. **J** Transwell assay detection of invasion and migration of HCC cells transfected with shNC or shKIAA1429-1/-2 (Scale bar = 100 μm). All experiments were repeated at least 3 times. Data are presented as the mean ± SD. Statistical significance was determined by Student’s *t*-test (for A) or one-way ANOVA (for C-J). *p < 0.05, **p < 0.01, ***p < 0.001.

KIAA1429 has been reported to play crucial roles in the development of various tumors, including HCC [17]. So far, whether KIAA1429 conferred HCC development through promoting immune evasion has not been clarified. Herein, western blotting was performed to analyze the expression of KIAA1429 at the protein level in 10 paired HCC specimens and normal tissues. We found that the expression of KIAA1429 was elevated in HCC (Fig. 3A). Moreover, the data obtained from TCGA database revealed that patients with higher KIAA1429 expression had a lower overall survival (Fig. 3B). Subsequently, the relationship between the abnormal high expression of KIAA1429 and clinicopathological parameters of 60 cases of HCC was analyzed. High KIAA1429 expression positively correlated with tumor size, but not patient age, sex, and TNM stage (Supplementary Table 2). Furthermore, up-regulation of KIAA1429 protein level was observed in HCC cells (Fig. 3C). Given that KIAA1429 was aberrantly up-regulated in HCC, we further explored its biological function in HCC cells. For this purpose, KIAA1429 was silenced in HCC cells by transfection with shKIAA1429-1 and shKIAA1429-2, and their interference efficiencies were validated via Western blotting (Fig. 3D). KIAA1429 has been suggested as a regulator of PD-L1 expression, which affects the tumor immune microenvironment of esophageal squamous cell carcinoma [23]. Thus, the regulation of KIAA1429 in PD-L1 expression in HCC cells was assessed by western blotting. We found that KIAA1429 deficiency markedly repressed PD-L1 expression in HCC cells (Fig. 3D). Furthermore, immunofluorescence staining was adopted to verify the above result. We found that expression of PD-L1 with green fluorescence was weakened in KIAA1429-silenced HCC cells (Fig. 3E). Additionally, knockdown of KIAA1429 or treatment with anti-PD-L1 increased CD8^+^ T cell percentages in the coculture system of PBMCs and HCC cells, which mimics the complexity of the tumor microenvironment and interacts with the immune system. Furthermore, the combination of KIAA1429 knockdown with PD-L1 antibody further enhanced T lymphocyte activation (Fig. 3F). The release of IFN-γ from cocultured PBMCs and HCC cells was significantly increased upon either PD-L1 antibody treatment or KIAA1429 knockdown. Cotreatment with PD-L1 antibody and shKIAA1429 further increased the level of IFN-γ (Fig. 3G). We further performed CCK-8, colony formation and Transwell assays to analyze the influence of KIAA1429 on HCC cell phenotypes. Silencing of KIAA1429 suppressed the growth of HCC cells (Fig. 3H, I) and impaired migration and invasion in HCC cells (Fig. 3J). Thus, KIAA1429 contributed to immune evasion, growth, migration, and invasion of HCC cells.

### KIAA1429 knockdown restrained HCC immune evasion in vivo

**Fig. 4:**
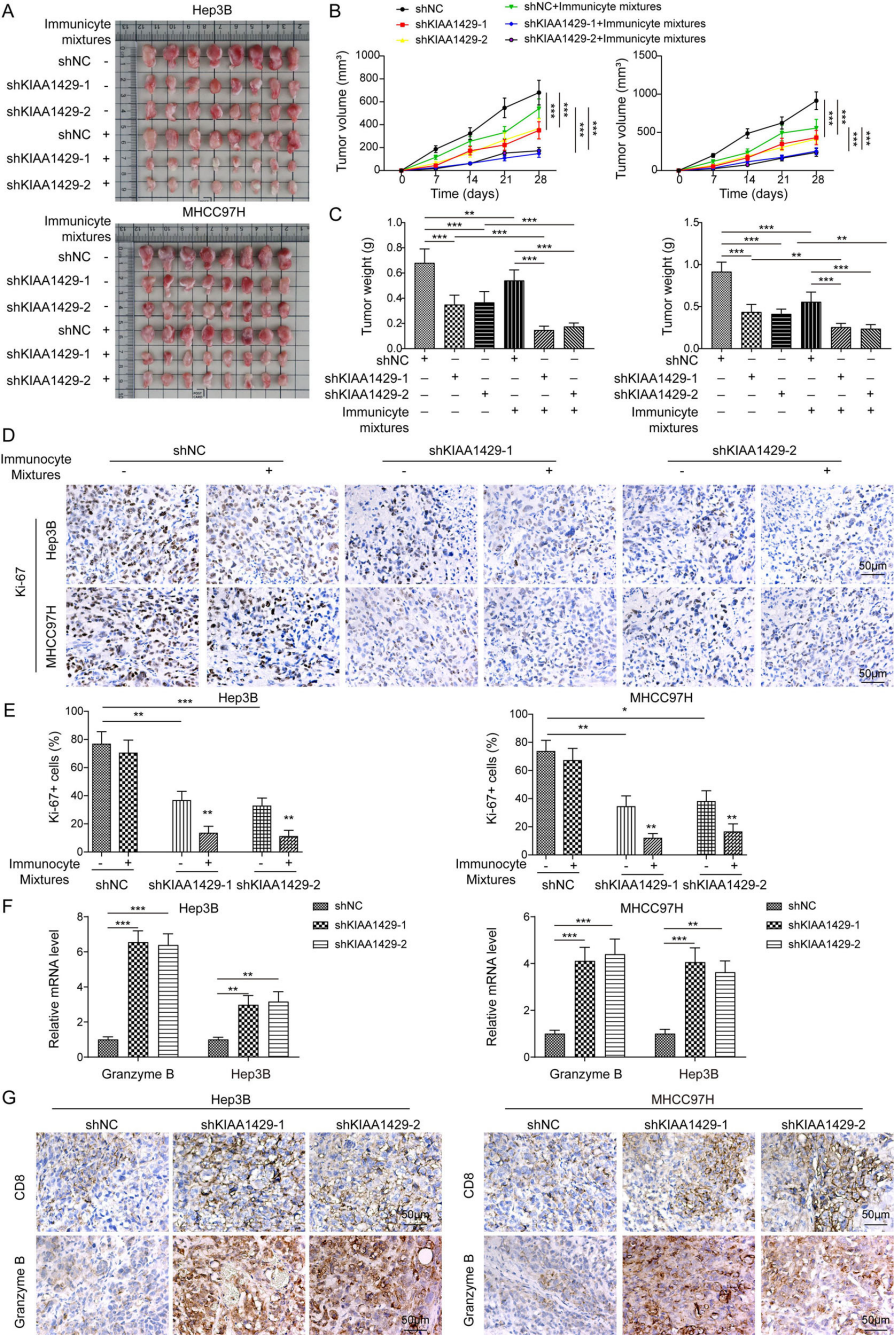
KIAA1429 knockdown restrained immune evasion and delayed HCC progression in vivo. NOD/SCID mice were subcutaneously injected with 2 × 10^6^ HCC cells infected with adenovirus-shNC or -shKIAA1429-1/-2. When the tumor volume was >100 mm^3^, the mice were injected with PBMCs (5 × 10^6^ /per mouse) combined activated T lymphocytes (1 × 10^7^ /per mouse) via tail intravenous once a week for three times. The growth of xenografts was monitored over 4 weeks. **A**–**C** Tumor images, tumor volume, and tumor weight were shown. **D** Immunohistochemistry staining analysis of Ki67 expression in tumor tissues (Scale bar = 50 µm). **E** Statistic analysis of percentage of Ki67 positive cells in tumor sections. **F** Granzyme B and IFN-γ expression in tumors was evaluated by RT-qPCR. **G** Immunohistochemistry staining analysis of granzyme B and CD8 expression in tumor tissues (Scale bar = 50 µm). *N* = 6. Data are presented as the mean ± SD. Statistical significance was determined by one-way ANOVA. *p < 0.05, **p < 0.01, ***p < 0.001.

To validate the anticancer effect of shKIAA1429 in vivo, a xenograft model in NOD/SCID mice was established. The results indicated that KIAA1429 depletion reduced the tumor weight and volume, which was enforced by cotreatment with immunocyte mixtures (Fig. 4A–C). We further detected the expression of Ki-67, a proliferation index, in tumor tissues via immunohistochemical staining. The result indicated that percentage of Ki-67 positive cells with brown staining was decreased after KIAA1429 knockdown, and this change was more obvious after cotreatment with immunocyte mixtures (Fig. 4D, E). Furthermore, the mRNA levels of T-cell markers CD8 and Granzyme B were measured by RT-qPCR. We found an increase in the expression of granzyme B and CD8 after KIAA1429 knockdown (Fig. 4F). Consistently, immunohistochemical staining showed that granzyme B and CD8 positive cells with brown staining were attenuated by KIAA1429 silencing (Fig. 4G). Altogether, these results indicated that KIAA1429 deficiency restrained immune evasion in vivo.

### TIP60-mediated KIAA1429 acetylation promoted HCC cell immune evasion

**Fig. 5:**
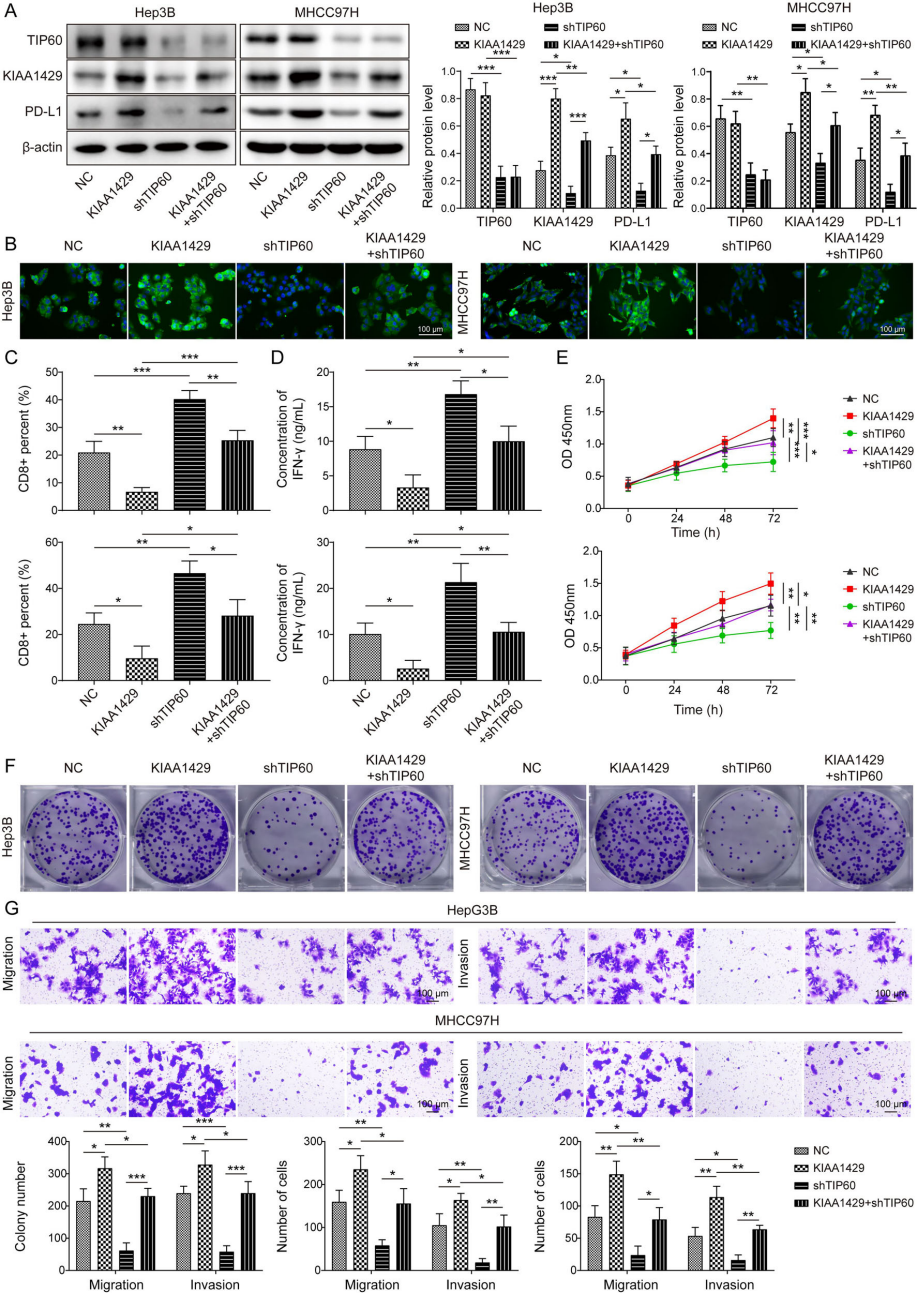
TIP60 promoted HCC cell immune evasion via KIAA1429 acetylation. Hep3B and MHCC97H cells were transfected with shTIP60, KIAA1429 plasmid, or a combination of them. **A** Western blotting analysis of TIP60, KIAA1429, and PD-L1 expression in HCC cells. **B** Immunofluorescence staining of PD-L1 expression (green) in Hep3B and MHCC97H cells after transfection (Scale bar = 50 µm). **C** Flow cytometry analysis of the proportion of CD8^+^ T cells in PBMCs co-cultured with HCC cells with various transfections. **D** ELISA measured IFN-γ level in the co-culture of HCC cells with various transfections and PBMCs. **E** CCK-8 and (**F**) colony formation assay determined the growth of HCC cells after various transfections. **G** HCC cell invasion and migration were detected by Transwell assay (Scale bar = 100 μm). All experiments were repeated at least 3 times. Data are presented as the mean ± SD. Statistical significance was determined by one-way ANOVA. *p < 0.05, **p < 0.01, ***p < 0.001.

To examine whether TIP60 contributed to HCC cell immune evasion through regulation of KIAA1429, HCC cells were transfected with KIAA1429 overexpression plasmid, shTIP60, or a combination of them. After transfection, the expression of TIP60, KIAA1429, and PD-L1 was assessed by western blotting. According to the data, KIAA1429 overexpression enhanced PD-L1 expression, but not affected TIP60 level. TIP60 knockdown reduced KIAA1429 and PD-L1 expression, and reversed KIAA1429 overexpression-mediated up-regulation of PD-L1 (Fig. 5A). Immunofluorescent staining was further adopted to examine PD-L1 expression. We found that PD-L1 expression with green fluorescence was enhanced by KIAA1429 overexpression, while TIP60 depletion inhibited PD-L1 expression and abolished KIAA1429 overexpression-mediated increased expression of PD-L1 (Fig. 5B). To analyze how KIAA1429 affect antitumor immunity of PBMCs to HCC cells, PBMCs were cocultured with HCC cells. We found that KIAA1429 overexpression lowered CD8^+^ T cell percentage and IFN-γ concentration, while TIP60 inhibition exerted the opposite roles and counteracted KIAA1429 overexpression-mediated the above changes (Fig. 5C, D). Functional experiments revealed that the proliferation, migration, and invasion of HCC cells were facilitated by KIAA1429 overexpression, which could be abrogated by shTIP60 co-transfection (Fig. 5E–G). To sum up, KIAA1429 was involved in TIP60-induced immune evasion during HCC progression.

### KIAA1429-mediated m^6^A modification promoted KDM5B expression

**Fig. 6:**
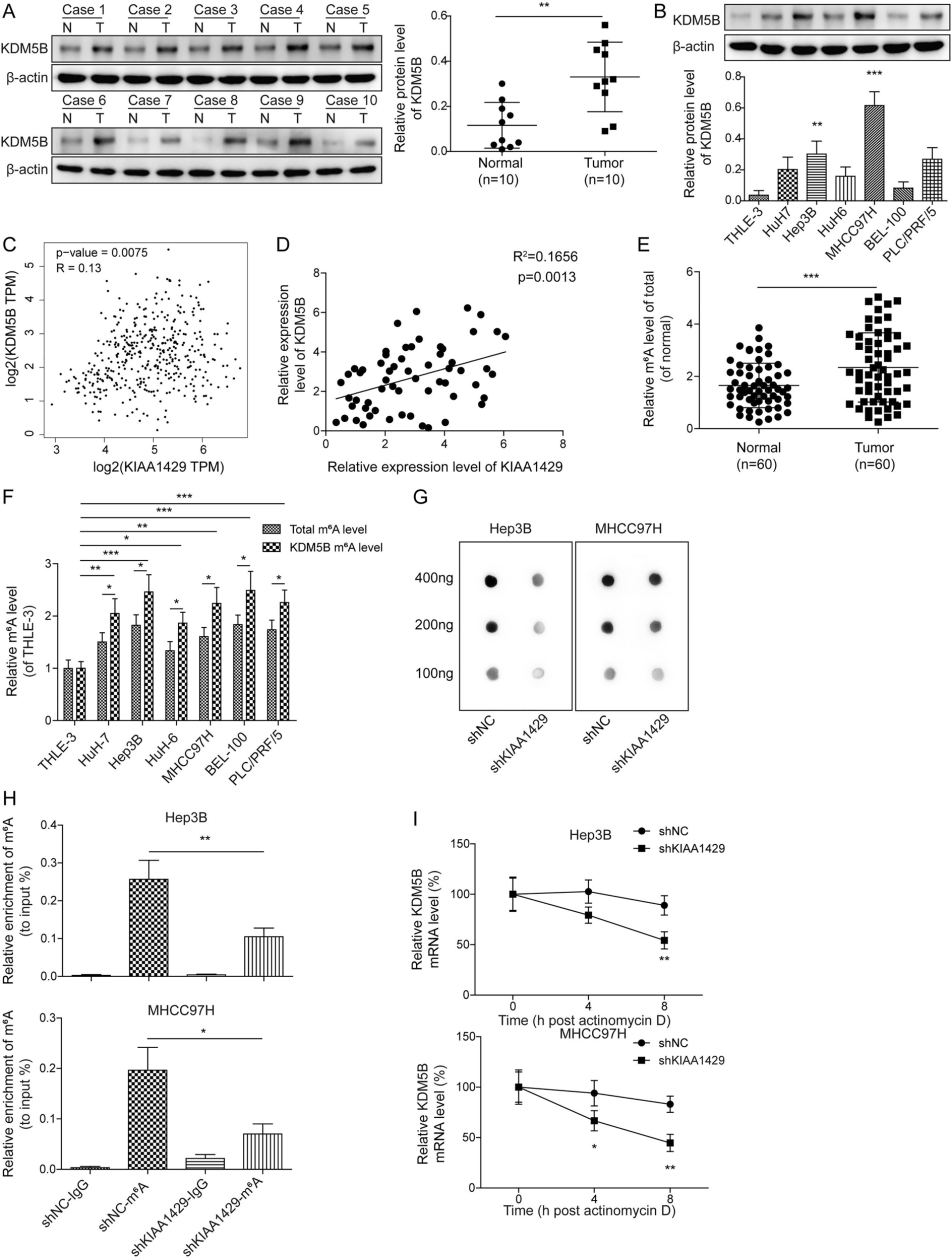
KIAA1429 enhanced KDM5B expression via m6A modification. **A** Western blotting analysis of KDM5B expression in HCC specimens and paired para-tumor tissues (*N* = 10). **B** KDM5B level in multiple HCC cell lines and normal THLE-3 cells was assessed by western blotting. **C,** **D** Correlation analysis of KIAA1429 and KDM5B expression in TCGA database and HCC samples. **E** Total m^6^A level in HCC samples and normal controls was determined via an RNA methylation quantification assay (*N* = 60). **F** Total and KDM5B m^6^A levels in HCC cells were assessed via RNA methylation quantification assay and MeRIP-qPCR, respectively. **G** Hep3B and MHCC97H cells were transfected with shNC or shKIAA1429 for 48 h. The m^6^A level in HCC cells was detected via an m^6^A dot blot. **H** MeRIP-qPCR analysis of the KDM5B m^6^A level in HCC cells transfected with shNC or shKIAA1429 for 48 h. **I** The stability of KDM5B mRNA in HCC cells transfected with shNC or shKIAA1429 was assessed after exposure to actinomycin D for 4, 8 h. All experiments were repeated at least 3 times. Data are presented as the mean ± SD. Statistical significance was determined by Student’s *t-*test (for A, E, H, I) or one-way ANOVA (for B, F). *p < 0.05, **p < 0.01, ***p < 0.001.

As KIAA1429 played a key role in HCC cell growth and invasion, we further investigated the downstream mechanisms underlying its function. KDM5B has been documented to contribute to self-renewal of HCC cells [17], and KDM5B could be modulated by m6A modification [24, 25]. According to RM2Target database (http://rm2target.canceromics.org/#/home), we selected 8 potential m6A writers for KDM5B, including METTL3, ZC3H13, ZCCHC4, WTAP, KIAA1429, RBMX, METTL16, and METTL14. Furthermore, the expression levels of these m6A writers were analyzed by GEPIA database. The data showed that KIAA1429 was significantly highly expressed, and ZC3H13 was significantly lowly expressed in HCC (supplementary Fig. 2A). There was an increasing trend of RBMX and ZCCHC4 expression in HCC, but there was no statistical difference (supplementary Fig. 2A). The expression of other m6A writers was not changed (supplementary Fig. 2A). Thus, KIAA1429, ZC3H13, RBMX, and ZCCHC4 were selected to evaluate their interaction with KDM5B by RNA-pull down assay. The results indicated that only KIAA1429 could bind to KDM5B mRNA (supplementary Fig. 2B). The binding of KIAA1429 to KDM5B was predicted by RM2Target database (supplementary Fig. 2C). Thus, we speculated that KDM5B expression might be affected by KIAA1429-mediated m6A modification in HCC. Western blotting demonstrated that KDM5B was highly expressed in HCC tissues (Fig. 6A) and cell lines (Fig. 6B) in comparison with normal controls. A positive correction between KDM5B and KIAA1429 expression was found in HCC in TCGA data (Fig. 6C), as well as in 60 HCC patients (Fig. 6D). Given that the dysregulation of KIAA1429 and KDM5B was closely associated with HCC, we further investigated whether KIAA1429 could regulate KDM5B expression in HCC. Initially, we observed a significant increase in the total m^6^A level in HCC samples (Fig. 6E). HCC cells consistently exhibited higher levels of total m^6^A and KDM5B m^6^A levels (Fig. 6F), suggesting the potential regulation of m^6^A in KDM5B. To investigated the role of m6A methyltransferase KIAA1429 in m^6^A modification of KDM5B, m^6^A dot blot assays and RNA m^6^A quantification were performed in HCC cells after transfection with shKIAA1429. The levels of m^6^A were significantly reduced in KIAA1429-silenced HCC cells (Fig. 6G). Furthermore, the MeRIP-qPCR assay revealed that KIAA1429 inhibition significantly reduced the enrichment of m^6^A modification in KDM5B (Fig. 6H). Then, Actinomycin D assay was performed to explore the effect of KIAA1429 depletion on the stability of KDM5B mRNA. Notably, knockdown of KIAA1429 markedly reduced the stability of KDM5B mRNA in response to actinomycin D exposure (Fig. 6I). Therefore, KDM5B mRNA stability was elevated by KIAA1429 via m^6^A modification.

### KIAA1429 promoted KDM5B expression in an m^6^A-YTHDF1-dependent manner

**Fig. 7:**
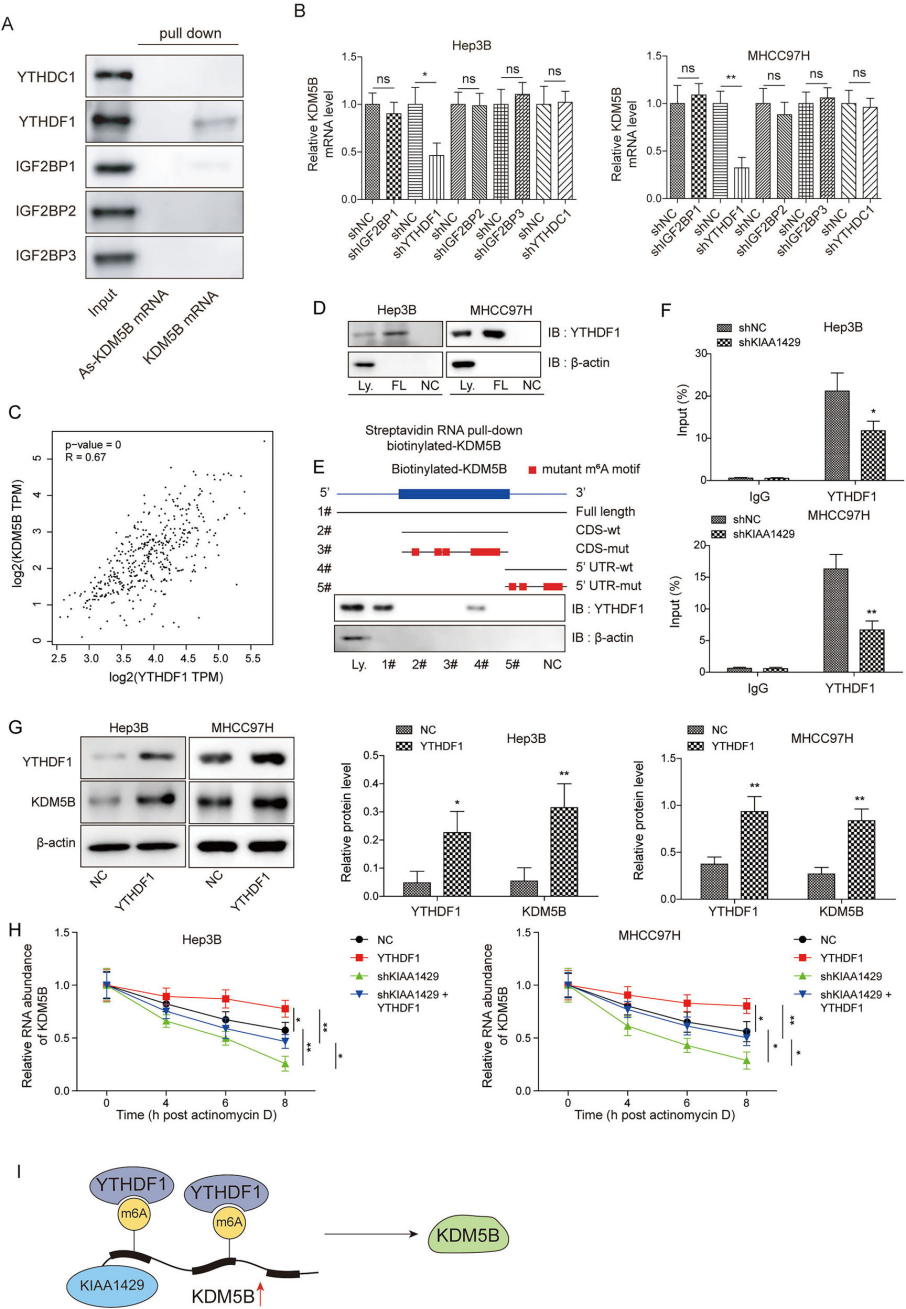
KIAA1429 promoted KDM5B expression in an m^6^A-YTHDF1-dependent manner. **A** RNA pulldown evaluated the binding of YTHDC1, YTHDF1, and IGF2BP1-3 to KDM5B. **B** Hep3B and MHCC97H cells were transfected with shNC, shYTHDC1, shYTHDF1, or shIGF2BP1-3 for 48 h. KDM5B expression in HCC cells was assessed by RT-qPCR after transfection. **C** Correlation analysis of YTHDF1 and KDM5B expression according to TCGA data. **D**, **E** Western blotting analysis of YTHDF1 after RNA pulldown. **F** RIP assay validation of the direct binding between the YTHDF1 and KDM5B mRNA. (**G**) Western blotting analysis of YTHDF1 and KDM5B levels in Hep3B and MHCC97H cells transfected with vector or YTHDF1 plasmid for 48 h. **H** KDM5B mRNA stability was assessed in Hep3B and MHCC97H cells transfected with vector, YTHDF1 plasmid, shKIAA1429, or YTHDF1 plasmid plus shKIAA1429 after exposure to actinomycin D for 4, 6, 8 h. **I** A flow chart of the results. All experiments were repeated at least 3 times. Data are presented as the mean ± SD. Statistical significance was determined by Student’s *t*-test (for B, F, G) or one-way ANOVA (for H). *p < 0.05, **p < 0.01, ***p < 0.001.

Since the m^6^A reader proteins YTHDC1, YTHDF1, and IGF2BP1-3 were predicted to bind to KDM5B, we assessed whether theses m^6^A reader proteins affected the KDM5B mRNA expression. We have performed RNA-pull down assay to evaluate the binding of YTHDC1, YTHDF1, and IGF2BP1-3 to KDM5B. The results showed that only YTHDF1 could bind to KDM5B mRNA (Fig. 7A). Besides, YTHDF1 knockdown resulted in the largest decrease in KDM5B mRNA (Fig. 7B). Based on these results, YTHDF1 was focused on in the subsequent experiments. Moreover, TCGA data analysis indicated a positive correlation between YTHDF1 and KDM5B expression (Fig. 7C). An RNA pulldown assay indicated that YTHDF1 bound to the full-length transcript of KDM5B (Fig. 7D). To further analyze the exact binding region of KDM5B, the biotinylated KDM5B probes targeting full length, coding sequence (CDS), and 5′-untranslated region (5′-UTR) regions were adopted. Notably, YTHDF1 predominantly bound to 5′-UTR of KDM5B, rather than the CDS in HCC cells (Fig. 7E). RIP assay was adopted to further validate the interaction between YTHDF1 and KDM5B, and we found that KIAA1429 depletion significantly weakened the binding between YTHDF1 and KDM5B (Fig. 7F). Next, the modulation of YTHDF1 in KDM5B expression was determined. Overexpression of YTHDF1 elevated the protein level of KDM5B in HCC cells (Fig. 7G). Furthermore, actinomycin D assay showed that knockdown of KIAA1429 enhanced the degradation of KDM5B mRNA, whereas the overexpression of YTHDF1 improved the stability of KDM5B mRNA, partially restoring KDM5B mRNA degradation affected by KIAA1429 silencing (Fig. 7H). Overall, these results demonstrated that KIAA1429 promoted m^6^A modification of KDM5B via recognition by YTHDF1, thereby maintaining the stability of KDM5B mRNA and enhancing its expression (Fig. 7I).

### Downregulated KDM5B expression repressed PD-L1-mediated immune evasion, growth, migration, and invasion of HCC cells

Since KIAA1429 contributed to KDM5B expression, we then investigated the biological function of KDM5B in HCC cells. To achieve this, HCC cells were transfected with shKDM5B. Western blotting indicated that PD-L1 expression was significantly reduced in KDM5B-silenced HCC cells (supplementary Fig. 3A). Immunofluorescent staining further demonstrated that KDM5B depletion reduced PD-L1 expression with green fluorescence (supplementary Fig. 3B). Furthermore, we analyzed the regulation of KDM5B in antitumor immunity in a coculture system of PBMCs and HCC cells. PD-L1 antibody pulldown or KDM5B deficiency raised CD8^+^ T cell percentage and IFN-γ release in the coculture of PBMCs and HCC cells, with a greater effect observed with PD-L1 antibody pulldown combined with KDM5B silencing (supplementary Fig. 3C, D). In addition, the growth, migration, and invasion of HCC cells were impaired after KDM5B knockdown (supplementary Fig. 3E–G). These observations supported the promotive role of KDM5B in immune evasion, proliferation, migration, and invasion of HCC cells.

### KIAA1429/YTHDF1 axis modulated immune evasion, growth, migration, and invasion of HCC cells

To investigate the role of KIAA1429/YTHDF1 axis in HCC cell progression, HCC cells were transfected with the YTHDF1 overexpression plasmid together with or without shKIAA1429. The changes in protein levels of KIAA1429, YTHDF1, KDM5B, and PD-L1 were assessed by western blotting after transfection. YTHDF1 overexpression increased the expression of YTHDF1, KDM5B, and PD-L1. However, the level of KIAA1429 was not affected. Conversely, KIAA1429 deficiency inhibited the expression of KDM5B, and PD-L1 with green fluorescence, and reversed YTHDF1 overexpression-mediated up-regulation of KDM5B and PD-L1 in HCC cells (supplementary Fig. 4A, B). Moreover, analysis of antitumor immunity in a coculture system of PBMCs and HCC cells indicated that YTHDF1 overexpression resulted in a decreased proportion of CD8^+^ T cells and IFN-γ release. However, KIAA1429 knockdown partly counteracted these changes (supplementary Fig. 4C, D). As expected, the HCC cell proliferation, migration, and invasion were enhanced with the overexpression of YTHDF1, which could be attenuated by KIAA1429 silencing (supplementary Fig. 4E–G). These findings suggested that YTHDF1 participated in KIAA1429-mediated immune evasion, growth, migration, and invasion of HCC cells.

### KIAA1429 favored immune evasion of HCC cells through KDM5B-mediated transcriptional inhibition of FoxO1

**Fig. 8:**
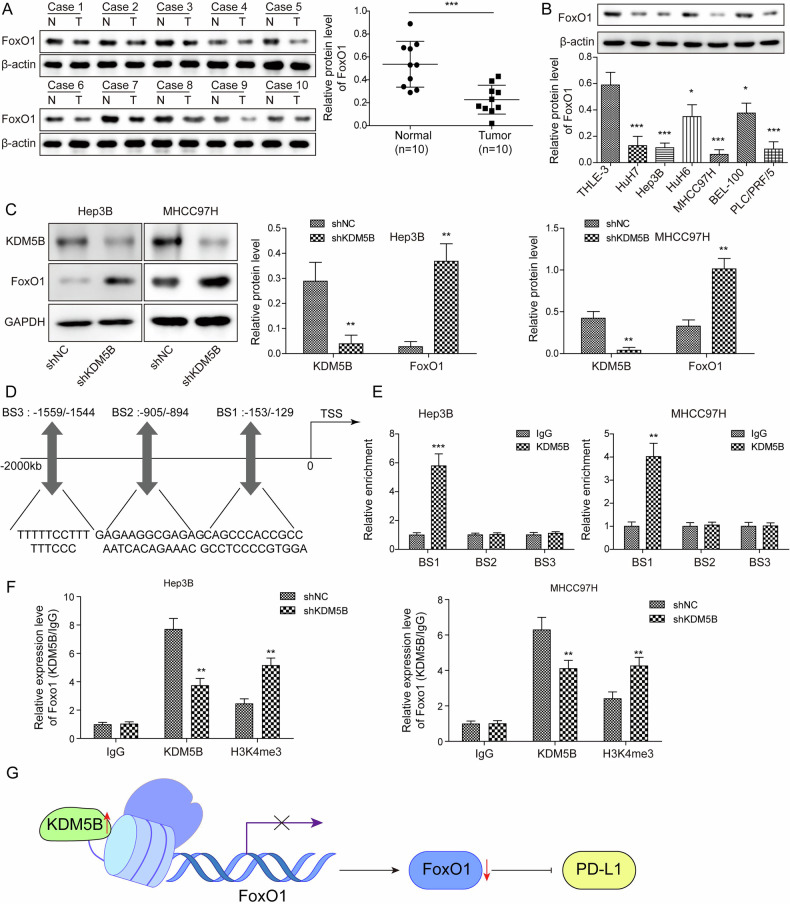
KDM5B caused transcriptional inhibition of FoxO1. **A** Western blotting analysis of FoxO1 expression in HCC specimens and paired para-tumor tissues (*N* = 10). **B** FoxO1 levels in HCC cells and normal THLE-3 cells were measured by western blotting. **C** Hep3B and MHCC97H cells were transfected with shNC or shKDM5B for 48 h. KDM5B and FoxO1 levels in HCC cells were assessed via western blotting. **D** AnimalTFDB4.0 database predicted the binding sites of KDM5B in the FoxO1 promoter. **E** ChIP assay validated the binding of KDM5B to the FoxO1 promoter. **F** The direct binding of KDM5B/H3K4me3 to the FoxO1 promoter in Hep3B and MHCC97H cells transfected with shNC or shKDM5B was assessed via ChIP. **G** A flow chart of the results. All experiments were repeated at least 3 times. Data are presented as the mean ± SD. Statistical significance was determined by Student’s *t*-test (for A, C, E, F) or one-way ANOVA (for B). *p < 0.05, **p < 0.01, ***p < 0.001.

KDM5B, a histone demethylase, can specifically decrease the level of H3K4me3, thereby modulating the expression of target genes [26]. Therefore, we investigated the downstream target genes of KDM5B during the development of HCC. As assessed by Western blotting, FoxO1 was down-regulated in HCC samples (Fig. 8A) and cell lines (Fig. 8B). Subsequently, the influence of KDM5B on FoxO1 expression in HCC cells was analyzed by western blotting. Increased expression of FoxO1 was validated in KDM5B-depleted HCC cells (Fig. 8C). To evaluate the interaction between KDM5B and FoxO1, AnimalTFDB4.0 database was adopted. The prediction data indicated that KDM5B may directly bind to FoxO1 at three binding sites (BS1, BS2, and BS3) (Fig. 8D). To validate the binding of KDM5B to FoxO1 promoter, ChIP assay was conducted. The results revealed that KDM5B directly bound to the BS1 site (-153/-129) in the FoxO1 promoter (Fig. 8E). Additionally, KDM5B depletion weakened the binding of KDM5B/H3K4me3 to the FoxO1 promoter (Fig. 8F). Thus, we concluded that KDM5B transcriptionally inhibited FoxO1 expression (Fig. 8G). To investigate the involvement of FoxO1 in KIAA1429-mediated malignant phenotype, HCC cells were transfected with shFoxO1, shKIAA1429, or a combination of both. The silencing efficiency of shFoxO1 was validated by western blotting (supplementary Fig. 5A). Western blotting and immunofluorescent staining further investigated the regulation of KIAA1429/FoxO1 axis in PD-L1 expression. FoxO1-silenced cells exhibited decreased FoxO1 and increased PD-L1 expression with green fluorescence, while the expression of KIAA1429 and KDM5B remained unaffected. Co-transfection with shKIAA1429 reversed the PD-L1 up-regulation and FoxO1 down-regulation caused by shFoxO1 transfection (supplementary Fig. 5B, C). For the evaluation of antitumor immunity in the HCC cell and PMBC coculture, FoxO1 silencing suppressed the proportion of CD8^+^ T cells and IFN-γ release. However, co-transfection with shKIAA1429 abolished these changes (supplementary Fig. 5D, E). Furthermore, shFoxO1-induced proliferation, migration, and invasion were impaired by KIAA1429 knockdown (supplementary Fig. 6A–C). Therefore, KIAA1429 contributed to immune evasion of HCC cells via KDM5B-mediated transcriptional inhibition of FoxO1.

## Discussion

**Fig. 9:**
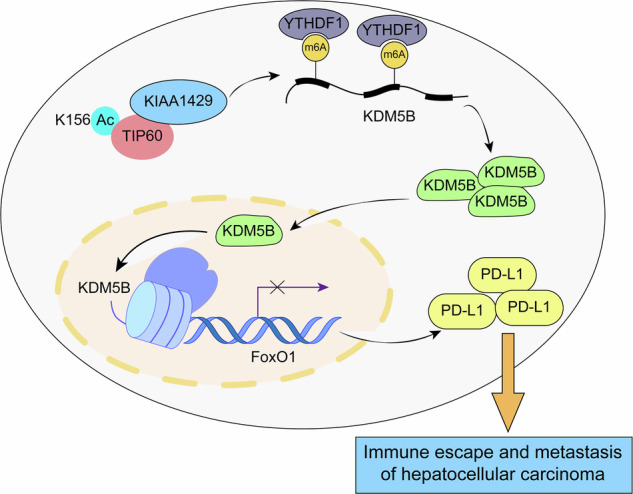
Mechanism diagram: TIP60-mediated acetylation of KIAA1429 enhanced KDM5B expression through m^6^A modification, which subsequently restrained transcription of FoxO1, thus favoring immune evasion, growth, migration, and invasion of HCC cells.

In recent years, immunotherapy blockade with PD-L1 has brought enormous benefit to the treatment of HCC. However, resistance to PD-L1 therapy often limits its success, causing poor prognoses in patients. Therefore, identifying the potential mechanisms of PD-L1 modulation in HCC could enhance therapy efficacy. The innovation of this study lines in the role of KIAA1429 in immune evasion mediated by PD-L1 during the development and metastasis of HCC and its related mechanisms. We for the first time discovered that TIP60 acetylated KIAA1429 to facilitate KDM5B expression through m^6^A modification, which consequently led to transcriptional inhibition of FoxO1, thereby contributing to the immune evasion, growth, migration, and invasion of HCC cells (Fig. 9). Our observations uncovered novel mechanisms of KIAA1429 underlying immune evasion and metastasis of HCC.

Increasing evidence has supported the hypothesis that oncogenes modulate the immune system, which is a crucial mechanism for tumor development [27]. HCC cells achieve immune escape through the interaction between PD-L1 and PD-1 that suppresses T cell proliferation and activity [28]. KIAA1429 has an oncogenic role in various cancers, including HCC [12, 29], and has been proposed as a biomarker for stomach adenocarcinomas as this is closely correlated with immune checkpoint molecules such as PD-L1 [30]. However, the regulation of KIAA1429 in HCC cell immune evasion has yet to be fully elucidated. Herein, we reported that the upregulated KIAA1429 expression was correlated with a poor prognosis in HCC. Interestingly, KIAA1429 knockdown reduced the expression of PD-L1, while enhancing the expression of IFN-γ and the proportion of CD8^+^ T cells, which consequently repressed HCC cell proliferation, migration, and invasion. Our findings demonstrated that KIAA1429 silencing repressed PD-L1-mediated immune escape to delay HCC progression.

To explore the possible upstream mechanism for the promotive role of KIAA1429 in immune escape of HCC cells, we focused on TIP60. TIP60 is one of acetyltransferases that exerts crucial roles in various biological events such as tumorigenesis and DNA repair [31]. Notably, TIP60 was reported to affect initiation and metastasis of HCC through acetylation of SPZ1-TWIST1 complex [32]. In line with this study, we demonstrated that KIAA1429 could be acetylated by TIP60, which was required for HCC cell immune evasion. We further explored the exact acetylation site of KIAA1429 and its functional significance. The acetylation level of KIAA1429 was reduced by the mutation of the K156 site, but not the K1587 site, which implied that KIAA1429 was acetylated at the K156 site. Functionally, abolishing the acetylation of KIAA1429 at the K156 site inhibited PD-L1, and TIP60-mediated KIAA1429 acetylation promoted PD-L1-mediated HCC cell immune evasion. Therefore, our results further explained how TIP60-mediated KIAA1429 acetylation at the K156 site facilitated HCC progression.

The m^6^A modification of RNA is recognized to play essential roles in the initiation and progression of various malignancies [33, 34]. Several studies have suggested that m6A modification, as well as diverse m6A regulatory factors, is implicated in innate immunity and anti-tumor responses [35, 36]. A recent study documented that m6A writer Wilms tumor 1-associated protein (WTAP) facilitated HCC immune evasion [37]. KIAA1429 was documented to function as an m^6^A regulator in various tumors. For example, KIAA1429 contributed to the development of diffuse large B-cell lymphoma by downregulating CHST11 expression via m6A modification [38]. A recent study reported that KIAA1429 promotes m^6^A methylation of FOXM1, leading to chemoresistance in gastric cancer [39]. Therefore, the identification of target molecules modulated by m^6^A is crucial in elucidating the regulatory mechanisms of KIAA1429 in HCC development. Herein, we predicted various m^6^A modification sites in KDM5B using the SRAMP database. KDM5B was demonstrated as an oncogene in diverse malignancies [40]. Zhang et al. suggested that KDM5B motivated immune evasion in melanoma via the recruitment of SETDB1 to inhibit retroelements [41]. We observed that KDM5B expression was upregulated in HCC and that KDM5B knockdown repressed immune evasion and metastasis. Furthermore, KDM5B expression was upregulated by KIAA1429 in an m^6^A-YTHDF1-dependent manner. Thus, KDM5B was demonstrated as a downstream target of KIAA1429. Notably, YTHDF1 overexpression markedly counteracted the inhibitory roles of KIAA1429 silencing in immune evasion and metastasis. Thus, KIAA1429 promotes immune evasion and metastasis in HCC through YTHDF recognition and m^6^A-mediated upregulation of KDM5B.

Next, to investigate the downstream mechanisms of KDM5B-mediated HCC progression, we analyzed the KDM5B histone demethylase function, which inhibits gene transcription and ultimately affects cell proliferation and differentiation [42]. KDM5B is a highly conserved member of the H3K4 demethylase family. A previous study documented that KDM5B facilitated the demethylation of H3K4me3 in the promoter region of METTL14, which repressed METTL14 transcription in NSCLC [43]. Our bioinformatics analysis showed that KDM5B can bind to the promoter region of FoxO1. FoxO1 is a forkhead box transcription factor involved in modulating apoptosis, antitumor immune regulation, and angiogenesis in various malignancies [44, 45]. Notably, FoxO1 plays a crucial role in maintaining CD8^+^ T cells-mediated tumor immunosurveillance [46]. Our previous study indicated that FoxO1 decreases PD-L1 level in a β-catenin-independent or -dependent manner to suppresses metastasis and immune evasion of HCC cells [47]. In this study, inhibition of KDM5B enhanced the enrichment of H3K4me3 in the FoxO1 promoter region. Thus, KDM5B directly bound to FoxO1 promoter to repress its transcription and expression in HCC cells in an H3K4me3-dependent manner. Moreover, inhibition of FoxO1 expression hampered the anticancer effects induced by KIAA1429 depletion. Therefore, we propose that KIAA1429 facilitates HCC cell immune evasion and metastasis through inducing KDM5B-mediated transcription inhibition of FoxO1.

Overall, we demonstrated that KIAA1429 functioned as an oncogene by contributing to immune evasion and metastasis of HCC cells. KIAA1429 was acetylated by TIP60 to enhance KDM5B mRNA stability and expression through m^6^A modification, which inhibited transcription of FoxO1. Our findings uncover the novel mechanisms of KIAA1429 in PD-L1-mediated immune evasion during HCC malignant development, which indicates targeting KIAA1429 as a possible intervention for HCC.

There are several limitations in this study. First, the sample size of patients with HCC was small, making it a challenge to draw robust conclusions. Second, immune escape cannot be conclusively demonstrated through in vitro experiments alone, and further evidence is needed to support immune escape. Furthermore, tumor tissues are also highly enriched with innate immune cells, such as myeloid-derived suppressor cells, tumor-associated macrophages, and tumor-associated neutrophils, which play critical roles in tumor immunity [48], but these innate immune cells were not assessed in the current study. These issues will be focused on in our future study.

## Conclusion

This study showed that the expression of TIP60, KIAA1429 and KDM5B was elevated, while FoxO1 expression was reduced in HCC specimens and cells. Depletion of TIP60, KIAA1429 or KDM5B was found to inhibit immune evasion and metastasis of HCC cells. Mechanistically, acetylation of KIAA1429 by TIP60 increased KDM5B expression in an m6A-dependent manner to transcriptionally repress FoxO1. Overall, our findings suggest that the TIP60/KIAA1429/KDM5B/FoxO1 axis is involved in immune evasion of HCC cells, and targeting this pathway may be a promising therapeutic strategy for treating HCC.

## Materials and methods

### Clinical sample

Clinical HCC tumor and peritumor control samples were collected from 60 cases of HCC during surgical excision from the Hunan Cancer Hospital. All patients signed written informed consents. This study was performed according to the guidelines of the Declaration of Helsinki and was approved by the Ethics Committee of Hunan Cancer Hospital.

### Cell culture

Hepatocyte cell line THLE-3 and HCC cell lines HuH-7, MHCC97H, PLC/PRF/5, and BEL-100 were provided by Shanghai Institutes of Biological Sciences, Chinese Academy of Sciences (Shanghai, China) and were cultured in MEM (Gibco, USA). Hep3B, HuH-6 HCC cells, and HEK293T cells were obtained from ATCC (USA) and were maintained in DMEM. All cells were supplemented with 10% fetal bovine serum (FBS, Gibco) and incubated at 37 °C with 5% CO_2_. All cell lines were authenticated by STR DNA profiling analysis and tested for mycoplasma contamination.

### Cell transfection

Small hairpin RNA (shRNA) targeting TIP60 (shTIP60-1 and shTIP60-2), KIAA1429 (shKIAA1429-1 and shKIAA1429-2), KDM5B (shKDM5B-1 and shKDM5B-2), FoxO1 (shFoxO1), IGF2BP1-3(shIGF2BP1-3), YTHDC1(shYTHDC1), YTHDF1 (shYTHDF1) and negative control shRNA (shNC), overexpression plasmids for YTHDF1 and KDM5B, Flag-KIAA1429-wild type (WT), Flag-KIAA1429 (K156R), Flag-KIAA1429 (K1587R), p-CMV4-TIP60, and Flag-CMV were provided by GenePharma (Shanghai, China). shRNA and plasmids were transfected into cells with Lipofectamine 2000 (Thermo Fisher, USA). Recombinant adenoviruses carrying shNC or shKIAA1429-1/-2 were packaged by GenePharma. HCC cells with 90% confluence were infected with the adenoviruses (10 × 10^9^ TU/mL).

### Real-time quantitative‐PCR

TRIzol reagent (Sigma-Aldrich, USA) was used to extract total RNA from HCC cells and tissues. cDNA was synthesized using a First Strand cDNA Synthesis Kit (Sigma-Aldrich). Real-time quantitative PCR (RT-qPCR) was performed using the SYBR® Green Quantitative RT-qPCR Kit (Sigma-Aldrich) and specific primers (Supplementary Table 1). The relative expression of genes normalized to GAPDH was calculated using the 2^-△△Ct^ method.

### Western blotting

Total protein was isolated using the RIPA lysis buffer (Elabscience, Wuhan, China) and quantified with the BCA Protein Colorimetric Assay Kit (Elabscience). Protein samples were separated via sodium dodecyl sulfate-polyacrylamide gel electrophoresis, followed by blotting onto polyvinylidene fluoride membranes and blocking for 1 h. Membranes were probed with the primary antibodies against KIAA1429 (1:500, 25712-1-AP, Proteintech, Wuhan, China), TIP60 (1:1000, ab300521, Abcam), KDM5B (1:1000, #15327, Cell Signaling Technology, USA), FoxO1 (1:1000, ab179450, Abcam), PD-L1 (1:1000, ab205921, Abcam), YTHDF1 (1:1000, ab220162, Abcam), acetyl‐lysine (Ac‐K; 1:1000, ab190479, Abcam), and β-actin (1:5000, #AF7018, Affinity Biosciences) overnight at 4 °C. After a 1-h incubation with the secondary antibody (1:2000, ab205718, Abcam), protein bands were visualized with the Excellent Chemiluminescent Substrate (ECL) Detection Kit (Elabscience). Full and uncropped western blots are shown in File named Original data.

### Immunofluorescence staining

4% paraformaldehyde-fixed HCC cells were permeabilized with 0.1% Triton X-100 and then blocked in 1% goat serum. HCC cells were then probed with anti-PD-L1 (1:500, ab205921, Abcam), anti-KIAA1429 (1:50, 25712-1-AP, Proteintech), anti-TIP60 (1:50, sc-166323, Santa Cruz, USA) at 4 °C overnight and subsequent secondary antibody for 1 h. Images were captured after nuclear staining with DAPI under a fluorescence microscope (Leica, Germany).

### Isolation of peripheral blood mononuclear cells and coculture with HCC cells

Peripheral blood mononuclear cells **(**PBMCs) were isolated from patients with HCC patients via Ficoll-Hypaque density gradient centrifugation. PBMCs were grown in RPMI 1640 with 10% FBS (Gibco) overnight. To activate T cells, suspended cells were harvested and stimulated with 10 μg/mL PHA and 4000 UI/mL rhIL-2 for 48 h. T cells were then cultured in RPMI 1640 with 2000 UI/mL rhIL-2 and 10% FBS. For coculture, HCC cells were plated into a 24-well plate and cultured in complete DMEM. PBMCs were placed in culture inserts (Corning, NY, USA) at 5 × 10^5^ cells/well, and cocultured with HCC cells for 48 h with or without PD-L1 blocking antibody (Sino Biological, Beijing, China).

### Flow cytometry

The percentage of CD8^+^ T cells in the coculture system of HCC cells and PBMCs were assessed via flow cytometry. After staining with anti-CD8 FITC (MHCD0801-4, eBiosciences), the cells were detected with a flow cytometer (Thermo Fisher).

### Enzyme-linked immunosorbent assay (ELISA)

The level of IFN-γ in the supernatant of the coculture system was assessed using the human IFN-γ ELISA Kit (Abcam).

### CCK-8 assay

HCC cells (3000) were seeded into 96-well plates, and CCK-8 reagent (10 μL, Yeasen, Shanghai, China) was added at different time points. After incubation for 2 h at 37 °C, the absorbance was measured at 450 nm on a microplate reader (Thermo Fisher).

### Colony formation

Colony formation was assessed to determine HCC cell growth. Briefly, 1000 cells were inoculated into a 6-well plate and cultured for two weeks. Resultant colonies were fixed in methanol and then stained with 0.1% crystal violet. Images were captured and quantified using ImageJ software.

### Transwell assay

Transwell chambers were precoated with or without Matrigel (Sigma-Aldrich). HCC cells were suspended in serum-depleted medium and added to the upper chambers while culture medium containing 10% FBS was added to lower chambers. Cells were allowed to migrate or invade for 24 h at 37 °C, followed by fixation in methanol and staining with 0.1% crystal violet. Cells were observed and quantified under a light microscope.

### Co-immunoprecipitation (Co-IP)

To verify the exogenous interaction between TIP60 and KIAA1429 proteins, 293 T cells were transfected with KIAA1429 and SFB-Flag-TIP60 plasmids. The transfected 293 T cells were lysed with IP lysis buffer (Beyotime, Haimen, China). Subsequently, immunoprecipitation with anti-SFB agarose beads was carried out. After rinsing, KIAA1429 and Flag protein abundance was detected by Western blotting.

To confirm the endogenous interaction between TIP60 and KIAA1429 proteins, as well as the acetylation level of KIAA1429, HCC cells were lysed with the IP lysis buffer, followed by pre‐cleaning with protein A/G beads at 4 °C for 4 h. Cell lysates were immunoprecipitated with the protein A/G beads conjugated with anti-TIP60 (ab300521, Abcam), anti-KIAA1429 (25712-1-AP, Proteintech), or Ac-K (ab190479; Abcam), or anti-IgG (ab172730, Abcam) at 4 °C overnight. Thereafter, the immunoprecipitated proteins were rinsed with IP lysis buffer and subjected to Western blotting.

### RNA m^6^A quantification

After isolation of total RNA using TRIzol reagent, the total m^6^A RNA level was assessed using the EpiQuik™ m^6^A RNA Methylation Quantification Kit (Epigentek, USA). Briefly, the purified RNA (200 ng) was seeded into assay wells and then incubated with the capture and detection antibodies, respectively. Finally, the absorbance was measured at 450 nm on a microplate reader, and the m^6^A level calculated by standard curve.

### Methylated RNA immunoprecipitation-qPCR

The methylated RNA immunoprecipitation (MeRIP) assay was performed on HCC cells using the Magna MeRIP™ m^6^A Kit (Millipore, USA). Briefly, extracted total RNA was substantially fragmented and incubated with m^6^A antibody (Millipore) or normal IgG conjugated to Protein A/G Magnetic Beads. The m^6^A-precipitated RNA was the eluted from the beads, and the relative enrichment of m^6^A was evaluated via qPCR.

### m^6^A dot blot assay

Total RNA or poly (A) + mRNA was prepared as previously reported [49]. The mRNA samples were added to 20 × SSC buffer (Sigma-Aldrich) at a ratio of 1:1 and reacted at 95 °C for 5 min. Subsequently, 100, 200, or 400 ng poly (A) + mRNA samples were spotted onto Hybond-N+ membranes (GE Healthcare, USA). Membranes were exposed to ultraviolet light for crosslinking of proteins and then blocked in 5% skim milk. The membrane was incubated with m^6^A antibody overnight at 4 °C, followed by incubation with secondary antibody and ECL development. RNA samples were also loaded to membranes for 0.02% methylene blue staining.

### RNA pulldown assay

RNA pulldown assays were performed using the Pierce Magnetic RNA-Protein Pull-Down Kit (Thermo Fisher). HCC cells were lysed in standard lysis buffer and then mixed with biotinylated RNA oligo probes conjugated to streptavidin beads at room temperature for 2 h. RNA-binding protein complexes were then eluted and detected via western blotting.

### RNA immunoprecipitation (RIP) assay

The Magna RIP™ RNA-Binding Protein Immunoprecipitation Kit (Millipore) was used to conduct the RIP assay. Prepared HCC cell lysates were incubated with magnetic beads precoated with anti-YTHDF1 (ab220162, 1:30, Abcam) or control IgG (ab172730, Abcam) overnight at 4 °C. After washing and treatment with proteinase K, RNA was isolated from RNA-protein complexes for RT-qPCR and agarose electrophoresis.

### Chromatin immunoprecipitation assay

The binding of KDM5B in FoxO1 promoter region was evaluated using chromatin immunoprecipitation (ChIP) with the Magna ChIP™ A/G kit (Millipore). HCC cells were fixed in 1% formaldehyde and then fragmented by ultrasonication. After centrifugation, the supernatant was mixed with Protein Agarose/Sepharose, followed by addition of anti-KDM5B (#15327, 1:50, Cell Signaling Technology), anti-H3K4me3 (#9751, 1:50, Cell Signaling Technology) or anti-IgG (ab172730, Abcam) and incubation at 4 °C overnight. After washing, DNA fragments were purified and assessed using RT-qPCR.

### GST pull-down assay

About 100 µg of GST-TIP60 fusion protein was incubated with 50 µL of glutathione agarose (Yeasen) for 1 h at 4 °C. Subsequently, the immobilized GST-TIP60 solutions were incubated with 100 µg of Flag-KIAA1429 fusion protein and GST at 4 °C overnight. Finally, the bound protein was eluted using 10 mM glutathione (pH 8.0) and detected by Western blotting.

### Animal models

This study was approved by the Ethics Committee of Hunan Cancer Hospital. Male 6-week-old NOD/SCID mice were obtained from Shanghai Experimental Animal Center of Chinese Academic of Sciences (Shanghai, China). Block pseudo-randomization was used for experimental group allocation. The investigators were blinded to grouping assignment. For the xenograft experiment, NOD/SCID mice were implanted with 2 × 10^6^ adenovirus-shNC or adenovirus-shKIAA1429-1/-2-infected HCC cells through subcutaneous injection. When the tumor volume was >100 mm^3^, mice were administered with human immunocyte mixtures that consisted of PBMCs (5 × 10^6^ /per mouse) and activated T lymphocytes (1 × 10^7^ /per mouse) or PBS via tail intravenous injection once a week for three times. Tumor volume was calculated by the equation: (length × width^2^)/2. After implantation, all mice were euthanized and tumors were obtained and weighed.

### Immunohistochemistry

After dewaxing, rehydration, and antigen recovery by citrate buffer, the paraffin-embedded tumor slices were incubated with primary antibodies: anti-Ki-67 (ab15580, 1:50), anti-CD8 (ab316778, 1:200) and anti-Granzyme B (ab255598, 1:200) (all antibodies were supplied by Abcam) at 4 °C overnight. Slices were then incubated with HRP‐conjugated secondary antibody and immuno-stained with DAB solution. Finally, the stained slices were observed using a light microscope.

### Statistical analysis

Sample size calculation was not conducted, while sample sizes were based on previous studies using xenograft model [50, 51]. All data are normally distributed (*P* > 0.05) analyzed by the Shapiro-Wilk test. Statistical analyses were performed using GraphPad Prism 8.0 software. Data from three independent experiments are presented as mean ± standard deviation (SD). One-way ANOVA followed by Bonferroni or Student’s *t*-test was performed for analysis of quantitative data. The parametric χ2 test was adopted for analyzing qualitative data. The Kaplan–Meier method was selected for survival analysis. The variance was similar between the groups and was statistically compared. A *P* < 0.05 was defined as significant.

## Supplementary information

Supplementary Materials

Original data

## Data Availability

All data generated or analysed during this study are included in this published article and its supplementary information files.

## References

[1] Bray F, Ferlay J, Soerjomataram I, Siegel RL, Torre LA, Jemal A. Global cancer statistics 2018: GLOBOCAN estimates of incidence and mortality worldwide for 36 cancers in 185 countries. CA Cancer J Clin. 2018;68:394–424. 30207593 10.3322/caac.21492

[2] Siegel RL, Miller KD, Jemal A. Cancer statistics, 2020. CA Cancer J Clin. 2020;70:7–30. 31912902 10.3322/caac.21590

[3] Okazaki T, Honjo T. PD-1 and PD-1 ligands: from discovery to clinical application. Int Immunol. 2007;19:813–24. 17606980 10.1093/intimm/dxm057

[4] Zou Y, Ye F, Kong Y, Hu X, Deng X, Xie J, et al. The Single-Cell Landscape of Intratumoral Heterogeneity and The Immunosuppressive Microenvironment in Liver and Brain Metastases of Breast Cancer. Adv Sci (Weinh). 2023;10:e2203699. 36529697 10.1002/advs.202203699 PMC9929130

[5] Nakano S, Eso Y, Okada H, Takai A, Takahashi K, Seno H. Recent Advances in Immunotherapy for Hepatocellular Carcinoma. Cancers (Basel). 2020;12:775. 32218257 10.3390/cancers12040775 PMC7226090

[6] Hargadon KM, Johnson CE, Williams CJ. Immune checkpoint blockade therapy for cancer: An overview of FDA-approved immune checkpoint inhibitors. Int Immunopharmacol. 2018;62:29–39. 29990692 10.1016/j.intimp.2018.06.001

[7] Qiao K, Chen C, Liu H, Qin Y. Pinin Induces Epithelial-to-Mesenchymal Transition in Hepatocellular Carcinoma by Regulating m6A Modification. J Oncol. 2021;2021:7529164. 34917148 10.1155/2021/7529164 PMC8670902

[8] Ping XL, Sun BF, Wang L, Xiao W, Yang X, Wang WJ, et al. Mammalian WTAP is a regulatory subunit of the RNA N6-methyladenosine methyltransferase. Cell Res. 2014;24:177–89. 24407421 10.1038/cr.2014.3 PMC3915904

[9] Ou X, Tan Y, Xie J, Yuan J, Deng X, Shao R, et al. Methylation of GPRC5A promotes liver metastasis and docetaxel resistance through activating mTOR signaling pathway in triple negative breast cancer. Drug Resist Updat. 2024;73:101063. 38335844 10.1016/j.drup.2024.101063

[10] Li Z, Zhang X, Liu C, Wu Y, Wen Y, Zheng R, et al. Engineering a Nano-drug Delivery System to Regulate m6A Modification and Enhance Immunotherapy in Gastric Cancer. Acta Biomater. 2024;191:412–27. 39581334 10.1016/j.actbio.2024.11.036

[11] Xu D, Liu Y, Liu Q, Li G, Zhang L, Yu C, et al. N(6)-methyladenosine modification of circular RNA circASH2L suppresses growth and metastasis in hepatocellular carcinoma through regulating hsa-miR-525-3p/MTUS2 axis. J Transl Med. 2024;22:1026. 39543614 10.1186/s12967-024-05745-z PMC11566831

[12] Lan T, Li H, Zhang D, Xu L, Liu H, Hao X, et al. KIAA1429 contributes to liver cancer progression through N6-methyladenosine-dependent post-transcriptional modification of GATA3. Mol Cancer. 2019;18:186. 31856849 10.1186/s12943-019-1106-z PMC6921542

[13] Janas JA, Zhang L, Luu JH, Demeter J, Meng L, Marro SG, et al. Tip60-mediated H2A.Z acetylation promotes neuronal fate specification and bivalent gene activation. Mol Cell. 2022;82:4627–46 e4614. 36417913 10.1016/j.molcel.2022.11.002 PMC9779922

[14] Liu R, Gou D, Xiang J, Pan X, Gao Q, Zhou P, et al. O-GlcNAc modified-TIP60/KAT5 is required for PCK1 deficiency-induced HCC metastasis. Oncogene. 2021;40:6707–19. 34650217 10.1038/s41388-021-02058-z PMC8677624

[15] Wang L, Long H, Zheng Q, Bo X, Xiao X, Li B. Circular RNA circRHOT1 promotes hepatocellular carcinoma progression by initiation of NR2F6 expression. Mol Cancer. 2019;18:119. 31324186 10.1186/s12943-019-1046-7 PMC6639939

[16] Gong J, Yan S, Yu H, Zhang W, Zhang D. Increased Expression of Lysine-Specific Demethylase 5B (KDM5B) Promotes Tumor Cell Growth in Hep3B Cells and is an Independent Prognostic Factor in Patients with Hepatocellular Carcinoma. Med Sci Monit. 2018;24:7586–94. 30353907 10.12659/MSM.910844 PMC6210936

[17] Guo JC, Liu Z, Yang YJ, Guo M, Zhang JQ, Zheng JF. KDM5B promotes self-renewal of hepatocellular carcinoma cells through the microRNA-448-mediated YTHDF3/ITGA6 axis. J Cell Mol Med. 2021;25:5949–62. 33829656 10.1111/jcmm.16342 PMC8256355

[18] Wang Y, Liu S, Li B, Liang J, Chen Y, Tang B, et al. KDM5B promotes SMAD4 loss-driven drug resistance through activating DLG1/YAP to induce lipid accumulation in pancreatic ductal adenocarcinoma. Cell Death Discov. 2024;10:252. 38789418 10.1038/s41420-024-02020-4 PMC11126577

[19] Lin X, Zuo S, Luo R, Li Y, Yu G, Zou Y, et al. HBX-induced miR-5188 impairs FOXO1 to stimulate beta-catenin nuclear translocation and promotes tumor stemness in hepatocellular carcinoma. Theranostics. 2019;9:7583–98. 31695788 10.7150/thno.37717 PMC6831466

[20] Chang Y, Zhou C, Fan L, Qiu G, Wang G, Wei G, et al. Upregulation of microRNA300 induces the proliferation of liver cancer by downregulating transcription factor FOXO1. Oncol Rep. 2018;40:3561–72. 30272296 10.3892/or.2018.6727

[21] Chi HC, Chen SL, Cheng YH, Lin TK, Tsai CY, Tsai MM, et al. Chemotherapy resistance and metastasis-promoting effects of thyroid hormone in hepatocarcinoma cells are mediated by suppression of FoxO1 and Bim pathway. Cell Death Dis. 2016;7:e2324. 27490929 10.1038/cddis.2016.227 PMC5108316

[22] Fueyo-Gonzalez F, Vilanova G, Ningoo M, Marjanovic N, Gonzalez-Vera JA, Orte A, et al. Small-molecule TIP60 inhibitors enhance regulatory T cell induction through TIP60-P300 acetylation crosstalk. iScience. 2023;26:108491. 38094248 10.1016/j.isci.2023.108491 PMC10716589

[23] Guo W, Tan F, Huai Q, Wang Z, Shao F, Zhang G, et al. Comprehensive Analysis of PD-L1 Expression, Immune Infiltrates, and m6A RNA Methylation Regulators in Esophageal Squamous Cell Carcinoma. Front Immunol. 2021;12:669750. 34054840 10.3389/fimmu.2021.669750 PMC8149800

[24] Jiao Y, Wang S, Wang X, Yin L, Zhang YH, Li YZ, et al. The m(6)A reader YTHDC2 promotes SIRT3 expression by reducing the stabilization of KDM5B to improve mitochondrial metabolic reprogramming in diabetic peripheral neuropathy. Acta Diabetol. 2023;60:387–99. 36574062 10.1007/s00592-022-01990-0

[25] Han Z, Xu Z, Yu Y, Cao Y, Bao Z, Gao X, et al. ALKBH5-mediated m(6)A mRNA methylation governs human embryonic stem cell cardiac commitment. Mol Ther Nucleic Acids. 2021;26:22–33. 34513291 10.1016/j.omtn.2021.05.019 PMC8408434

[26] Wang Z, Zhong C, Li H. Histone demethylase KDM5B catalyzed H3K4me3 demethylation to promote differentiation of bone marrow mesenchymal stem cells into cardiomyocytes. Mol Biol Rep. 2022;49:7239–49. 35788877 10.1007/s11033-022-07428-8 PMC9304058

[27] Casey SC, Baylot V, Felsher DW. MYC: Master Regulator of Immune Privilege. Trends Immunol. 2017;38:298–305. 28233639 10.1016/j.it.2017.01.002 PMC5378645

[28] Topalian SL, Drake CG, Pardoll DM. Targeting the PD-1/B7-H1(PD-L1) pathway to activate anti-tumor immunity. Curr Opin Immunol. 2012;24:207–12. 22236695 10.1016/j.coi.2011.12.009 PMC3319479

[29] Cheng X, Li M, Rao X, Zhang W, Li X, Wang L, et al. KIAA1429 regulates the migration and invasion of hepatocellular carcinoma by altering m6A modification of ID2 mRNA. Onco Targets Ther. 2019;12:3421–8. 31118692 10.2147/OTT.S180954 PMC6510231

[30] Mo P, Xie S, Cai W, Ruan J, Du Q, Ye J, et al. N(6)-methyladenosine (m(6)A) RNA methylation signature as a predictor of stomach adenocarcinoma outcomes and its association with immune checkpoint molecules. J Int Med Res. 2020;48:300060520951405. 32972288 10.1177/0300060520951405 PMC7522833

[31] Ghobashi AH, Kamel MA. Tip60: updates. J Appl Genet. 2018;59:161–8. 29549519 10.1007/s13353-018-0432-y

[32] Wang LT, Wang SN, Chiou SS, Liu KY, Chai CY, Chiang CM, et al. TIP60-dependent acetylation of the SPZ1-TWIST complex promotes epithelial-mesenchymal transition and metastasis in liver cancer. Oncogene. 2019;38:518–32. 30154425 10.1038/s41388-018-0457-z PMC6345675

[33] Chen XY, Zhang J, Zhu JS. The role of m(6)A RNA methylation in human cancer. Mol Cancer. 2019;18:103. 31142332 10.1186/s12943-019-1033-z PMC6540575

[34] Zou Y, Zheng S, Xie X, Ye F, Hu X, Tian Z, et al. N6-methyladenosine regulated FGFR4 attenuates ferroptotic cell death in recalcitrant HER2-positive breast cancer. Nat Commun. 2022;13:2672. 35562334 10.1038/s41467-022-30217-7 PMC9106694

[35] Li N, Hui H, Bray B, Gonzalez GM, Zeller M, Anderson KG, et al. METTL3 regulates viral m6A RNA modification and host cell innate immune responses during SARS-CoV-2 infection. Cell Rep. 2021;35:109091. 33961823 10.1016/j.celrep.2021.109091 PMC8090989

[36] Li B, Zhu L, Lu C, Wang C, Wang H, Jin H, et al. circNDUFB2 inhibits non-small cell lung cancer progression via destabilizing IGF2BPs and activating anti-tumor immunity. Nat Commun. 2021;12:295. 33436560 10.1038/s41467-020-20527-z PMC7804955

[37] Yu F, Feng Y, Wang Q, Sun J. N(6)-methyladenosine (m(6)A) Writer WTAP Potentiates Hepatocellular Carcinoma Immune Evasion and Aerobic Glycolysis. Cell Biochem Biophys. 2024;82:2321–31. 38872051 10.1007/s12013-024-01342-5

[38] Chen X, Lu T, Cai Y, Han Y, Ding M, Chu Y, et al. KIAA1429-mediated m6A modification of CHST11 promotes progression of diffuse large B-cell lymphoma by regulating Hippo-YAP pathway. Cell Mol Biol Lett. 2023;28:32. 37076815 10.1186/s11658-023-00445-w PMC10114474

[39] Tang B, Li M, Xu Y, Li X. N(6)-methyladenosine (m(6)A) writer KIAA1429 accelerates gastric cancer oxaliplatin chemoresistance by targeting FOXM1. J Cancer Res Clin Oncol. 2022;149:5037–45. 36326914 10.1007/s00432-022-04426-y PMC11797288

[40] Fu YD, Huang MJ, Guo JW, You YZ, Liu HM, Huang LH, et al. Targeting histone demethylase KDM5B for cancer treatment. Eur J Med Chem. 2020;208:112760. 32883639 10.1016/j.ejmech.2020.112760

[41] Zhang SM, Cai WL, Liu X, Thakral D, Luo J, Chan LH, et al. KDM5B promotes immune evasion by recruiting SETDB1 to silence retroelements. Nature. 2021;598:682–7. 34671158 10.1038/s41586-021-03994-2 PMC8555464

[42] Sun X, Li Z, Niu Y, Zhao L, Huang Y, Li Q, et al. Jarid1b promotes epidermal differentiation by mediating the repression of Ship1 and activation of the AKT/Ovol1 pathway. Cell Prolif. 2019;52:e12638. 31152465 10.1111/cpr.12638 PMC6797505

[43] Wang J, Wang S, Yang H, Wang R, Shi K, Liu Y, et al. Methyltransferase like-14 suppresses growth and metastasis of non-small-cell lung cancer by decreasing LINC02747. Cancer Sci. 2024;115:2931–46. 38888105 10.1111/cas.16254 PMC11462971

[44] Chen D, Yang Y, Yang P. Quxie Capsule Inhibits Colon Tumor Growth Partially Through Foxo1-Mediated Apoptosis and Immune Modulation. Integr Cancer Ther. 2019;18:1534735419846377. 31030593 10.1177/1534735419846377 PMC6488785

[45] Kim SY, Ko YS, Park J, Choi Y, Park JW, Kim Y, et al. Forkhead Transcription Factor FOXO1 Inhibits Angiogenesis in Gastric Cancer in Relation to SIRT1. Cancer Res Treat. 2016;48:345–54. 25761483 10.4143/crt.2014.247 PMC4720104

[46] Ginefra P, Hope HC, Chiang YH, Nutten S, Blum S, Coukos G, et al. Urolithin-A Promotes CD8+ T Cell-mediated Cancer Immunosurveillance via FOXO1 Activation. Cancer Res Commun. 2024;4:1189–98. 38626334 10.1158/2767-9764.CRC-24-0022 PMC11067828

[47] Xie W, Shi L, Quan H, Xiao H, Chen J, Liu J, et al. SYVN1 ubiquitinates FoxO1 to induce beta-catenin nuclear translocation, PD-L1-mediated metastasis, and immune evasion of hepatocellular carcinoma. Cell Oncol. (Dordr). 2023;46:1285–99. 10.1007/s13402-023-00811-y PMC10618324 37099251

[48] Matsuo K, Yoshie O, Nakayama T. Multifaceted Roles of Chemokines and Chemokine Receptors in Tumor Immunity. Cancers (Basel). 2021;13:6132. 34885241 10.3390/cancers13236132 PMC8656932

[49] Chen Y, Peng C, Chen J, Chen D, Yang B, He B, et al. WTAP facilitates progression of hepatocellular carcinoma via m6A-HuR-dependent epigenetic silencing of ETS1. Mol Cancer. 2019;18:127. 31438961 10.1186/s12943-019-1053-8 PMC6704583

[50] Luo Z, Lu L, Tang Q, Wei W, Chen P, Chen Y, et al. CircCAMSAP1 promotes hepatocellular carcinoma progression through miR-1294/GRAMD1A pathway. J Cell Mol Med. 2021;25:3793–802. 33484498 10.1111/jcmm.16254 PMC8051675

[51] Zhang T, Huang Y, Liu W, Meng W, Zhao H, Yang Q, et al. Overexpression of zinc finger protein 687 enhances tumorigenic capability and promotes recurrence of hepatocellular carcinoma. Oncogenesis. 2017;6:e363. 28737756 10.1038/oncsis.2017.63 PMC5541715

