# Supplementary material for: Acetylated KIAA1429 by TIP60 facilitates metastasis and immune evasion of hepatocellular carcinoma via N6-methyladenosine-KDM5B-mediated regulation of FoxO1
Source: Cell Death Discov. 2025 Apr 29;11:210. doi: 10.1038/s41420-025-02462-4 (PMC12041376; doi:10.1038/s41420-025-02462-4)
Supplement: Supplementary file 1 — Supplementary Materials [file 41420_2025_2462_MOESM1_ESM.pdf]

Supplementary Materials

1. Supplementary Figure and Figure legends

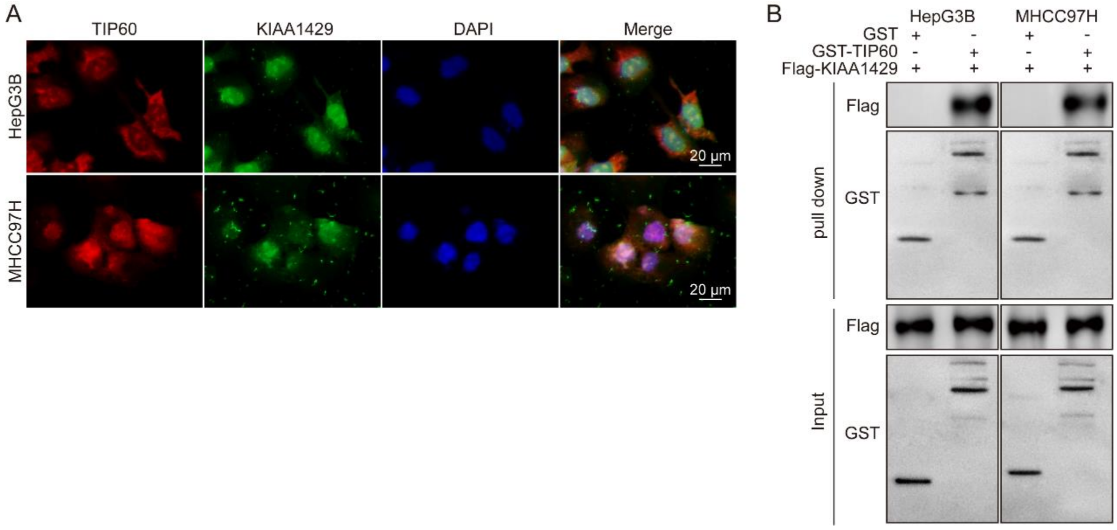

Supplementary Figure 1 The interaction between TIP60 and KIAA1429 in HCC cells. (A) Immunofluorescence staining evaluated the co-localization of KIAA1429 (green) and TIP60 (red) in HCC cells (Scale bar = 20 μm). Nuclei (blue) were stained with 4',6-diamidino-2-phenylindole (DAPI). (B) The binding of TIP60 to KIAA1429 was determined by GST pull-down assay. All experiments were repeated at least 3 times.

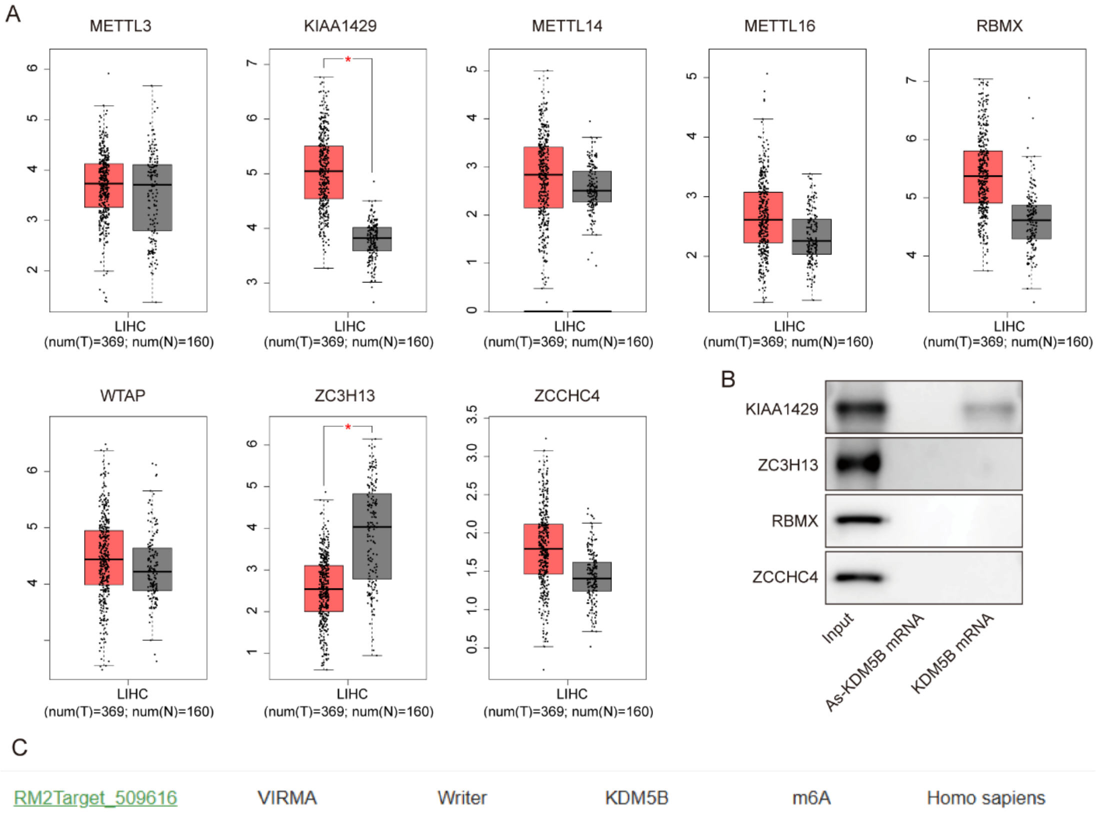

Supplementary Figure 2 KDM5B was a target gene for KIAA1429. (A) GEPIA database analyzed expression levels of METTL3, ZC3H13, ZCCHC4, WTAP, KIAA1429, RBMX, METTL16, and METTL14 in HCC and normal liver tissues. (B) The binding of KIAA1429, ZC3H13, RBMX, ZCCHC4 to KDM5B mRNA was assessed by RNA pulldown assay. (C) RM2Target database predicted the interaction between KIAA1429 and KDM5B. All experiments were repeated at least 3 times. *$p < 0.05$.

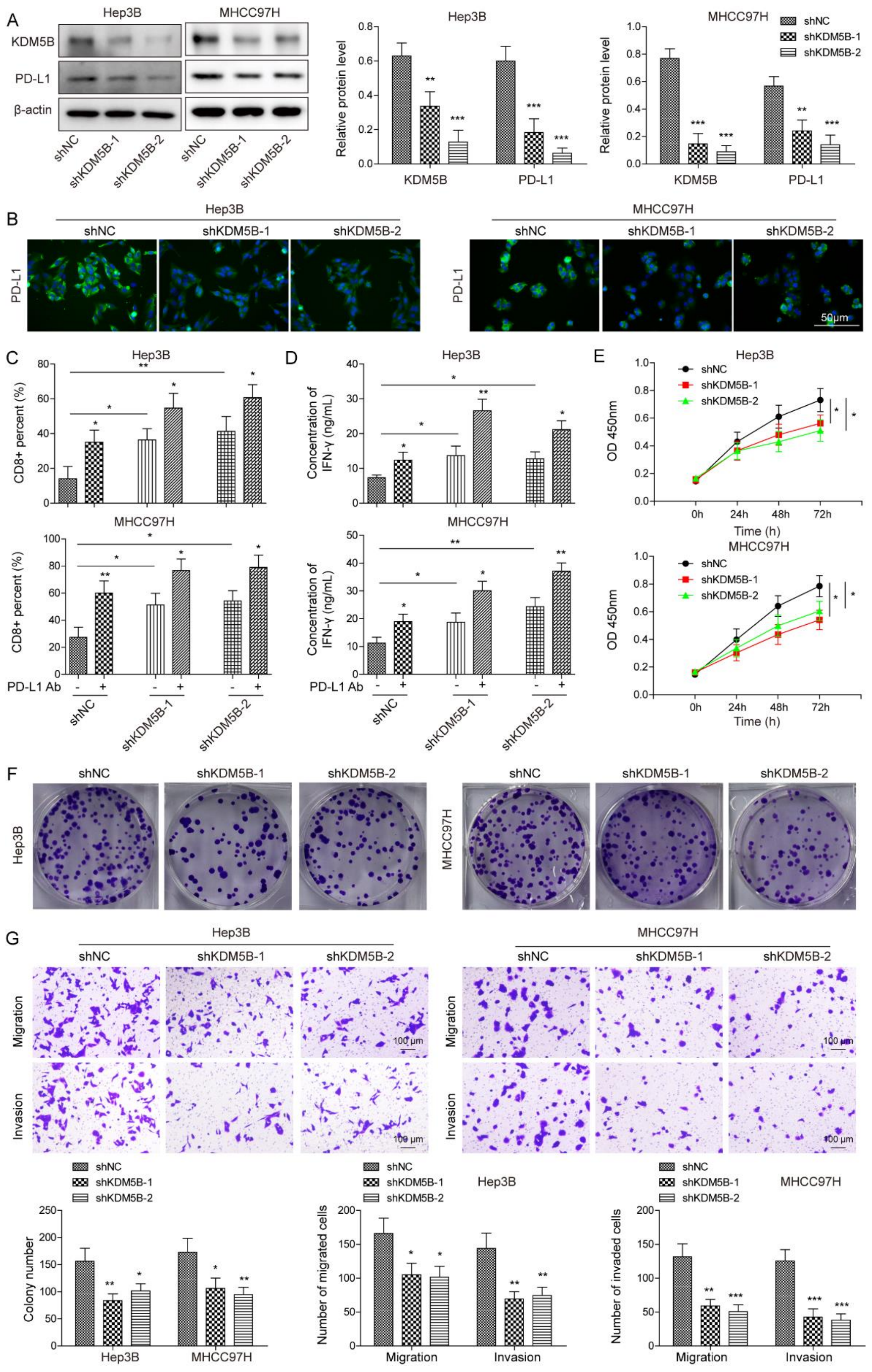

Supplementary Figure 3 KDM5B knockdown inhibited PD-L1-mediated immune evasion, growth, migration, and invasion of HCC cells. HCC cells were transfected

with shKDM5B-1, -2. (A) Western blotting analysis of KDM5B and PD-L1 expression in KDM5B-knockdown cells. (B) Immunofluorescence staining of PD-L1 in HCC cells (Scale bar = 50 μm). (C) Flow cytometry assessment of $CD8^+$ T cell proportion in HCC cells co-cultured with PBMCs detected. (D) ELISA assessment of IFN-γ level in the co-culture of HCC cells and PBMCs. (E) CCK-8 and (F) colony formation assay detected the proliferative ability of HCC cells. (G) Transwell assay analysis of HCC cell migration and invasion (Scale bar = 100 μm). All experiments were repeated at least 3 times. *$p < 0.05$, **$p < 0.01$, ***$p < 0.001$.

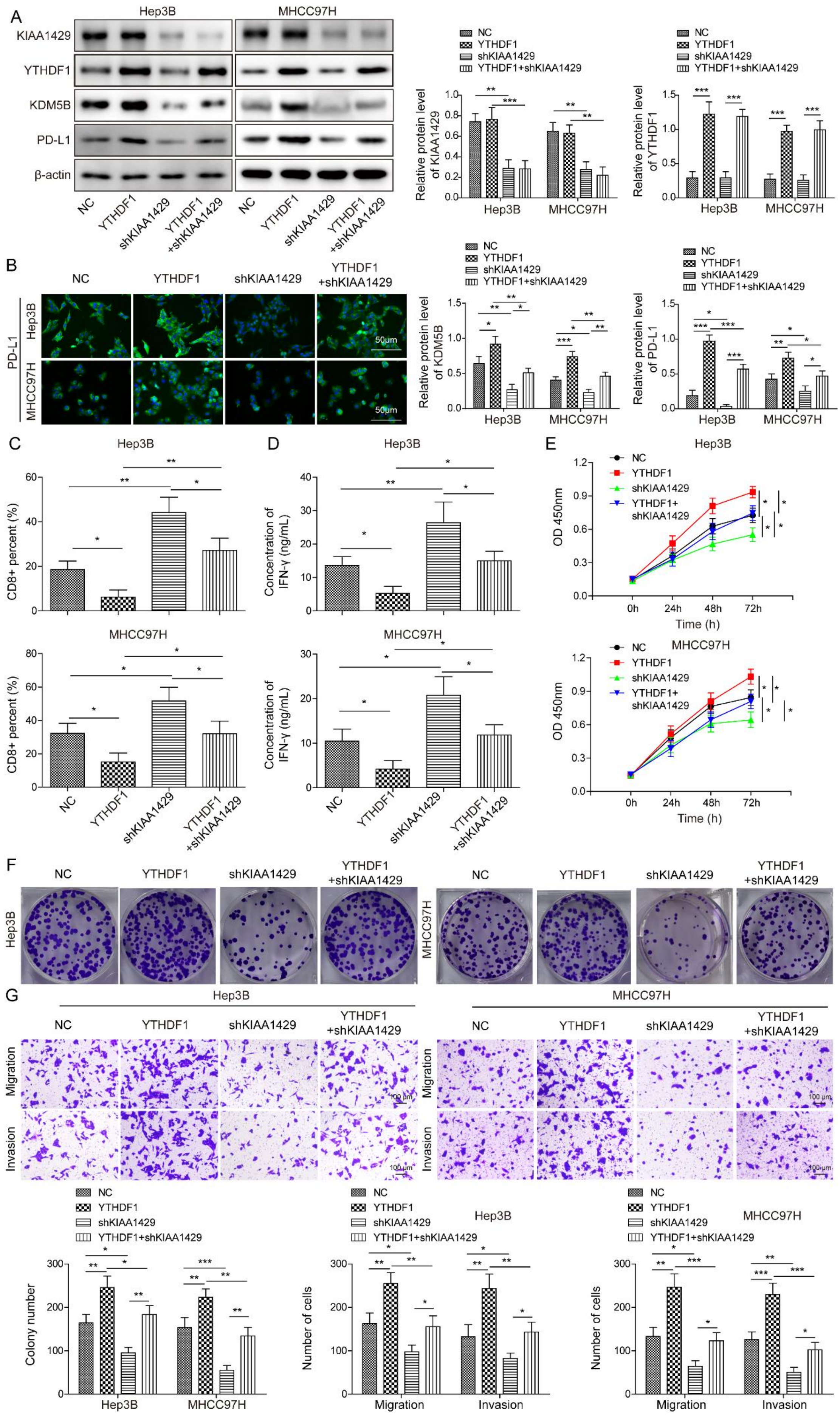

A
Hep3B
MHCC97H
KIAA1429
YTHDF1
KDM5B
PD-L1
β-actin
NC
YTHDF1
shKIAA1429
YTHDF1
+shKIAA1429
Relative protein level of KIAA1429
Relative protein level of YTHDF1
YTHDF1+shKIAA1429
B
PD-L1
50μm
Relative protein level of KDM5B
Relative protein level of PD-L1
C
CD8+ percent (%)
D
Concentration of IFN-γ (ng/mL)
E
OD 450nm
Time (h)
0h
24h
48h
72h
F
G
Migration
Invasion
100 μm
Colony number
Number of cells

Supplementary Figure 4 KIAA1429 promoted immune evasion, growth, migration, and invasion of HCC cells through modulation of YTHDF1. (A) Western blotting analysis of KIAA1429, YTHDF1, KDM5B, and PD-L1 levels in HCC cells transfected with shKIAA1429, YTHDF1 plasmid, or a combination of them. (B) PD-L1 expression (green) in HCC cells transfected with shKIAA1429, YTHDF1 plasmid, or a combination of them was evaluated by immunofluorescence staining (Scale bar = 50 μm). (C) Flow cytometry assessment of CD8+ T cell proportion in the co-culture system of PBMCs and HCC cells transfected with shKIAA1429, YTHDF1 plasmid, or a combination of them. (D) ELISA assessment of IFN-γ level in the co-culture system of PBMCs and HCC cells transfected with shKIAA1429, YTHDF1 plasmid, or a combination of them. (E) CCK-8 and (F) colony formation assay detected the proliferative ability of HCC cells transfected with shKIAA1429, YTHDF1 plasmid, or a combination of them. (G) Transwell assay analysis of migration and invasion of HCC cells transfected with shKIAA1429, YTHDF1 plasmid, or a combination of them (Scale bar = 100 μm). All experiments were repeated at least 3 times. Data are presented as the mean ± SD. Statistical significance was determined by one-way ANOVA. *$p < 0.05$, **$p < 0.01$, ***$p < 0.001$.

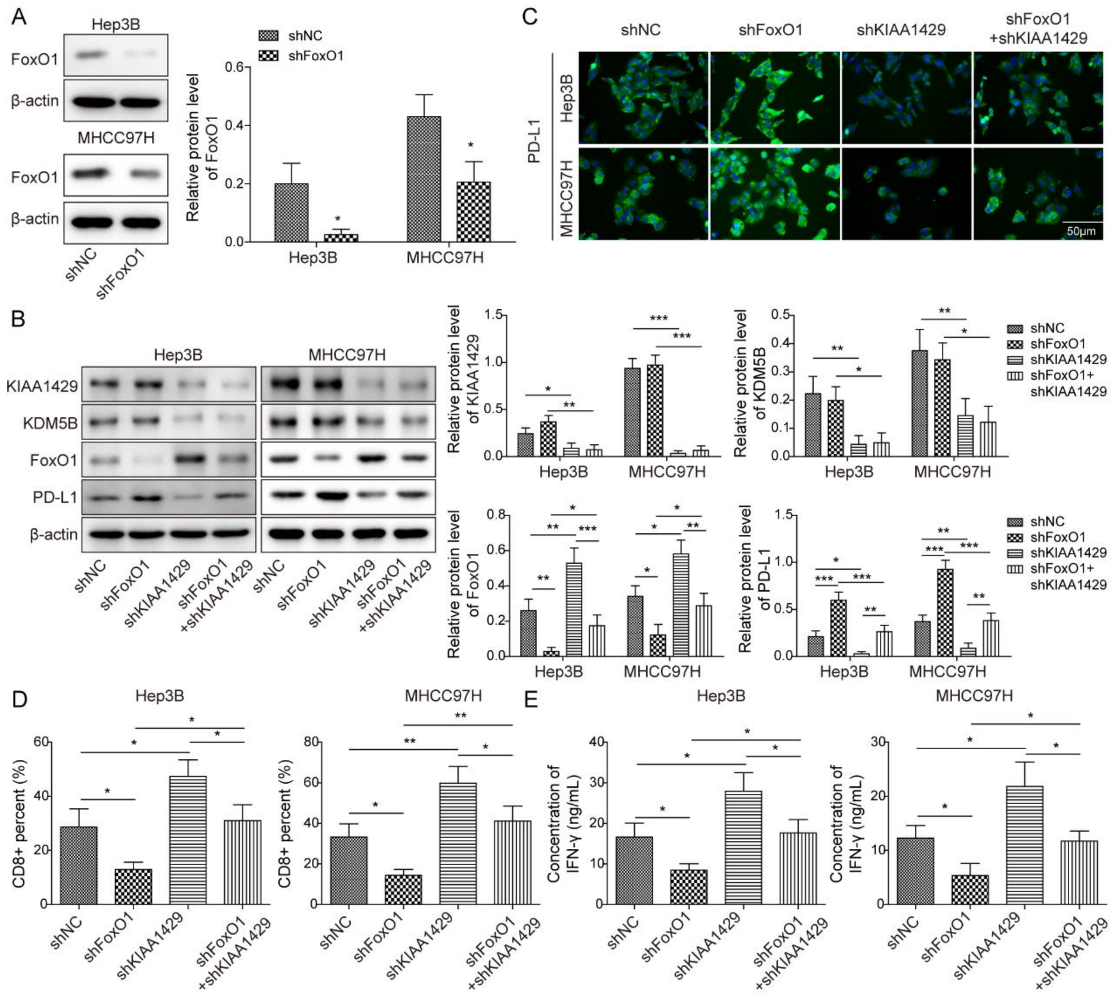

Supplementary Figure 5 FoxO1 down-regulation weakened the inhibitory effects of KIAA1429 deficiency on HCC cell immune evasion. HCC cells were transfected with shFoxO1, shKIAA1429, or a combination of them. (A) FoxO1 protein levels were measured via western blotting. (B) KIAA1429, KDM5B, FoxO1, and PD-L1 protein levels were measured via western blotting. (C) PD-L1 expression was observed via immunofluorescence staining (Scale bar = 50 μm). (D) Flow cytometry assessment of CD8^{+}T cell proportion in the co-culture system. (E) ELISA assessment of IFN-γ level in the co-culture system. All experiments were repeated at least 3 times. *$p < 0.05$, **$p < 0.01$, ***$p < 0.001$.

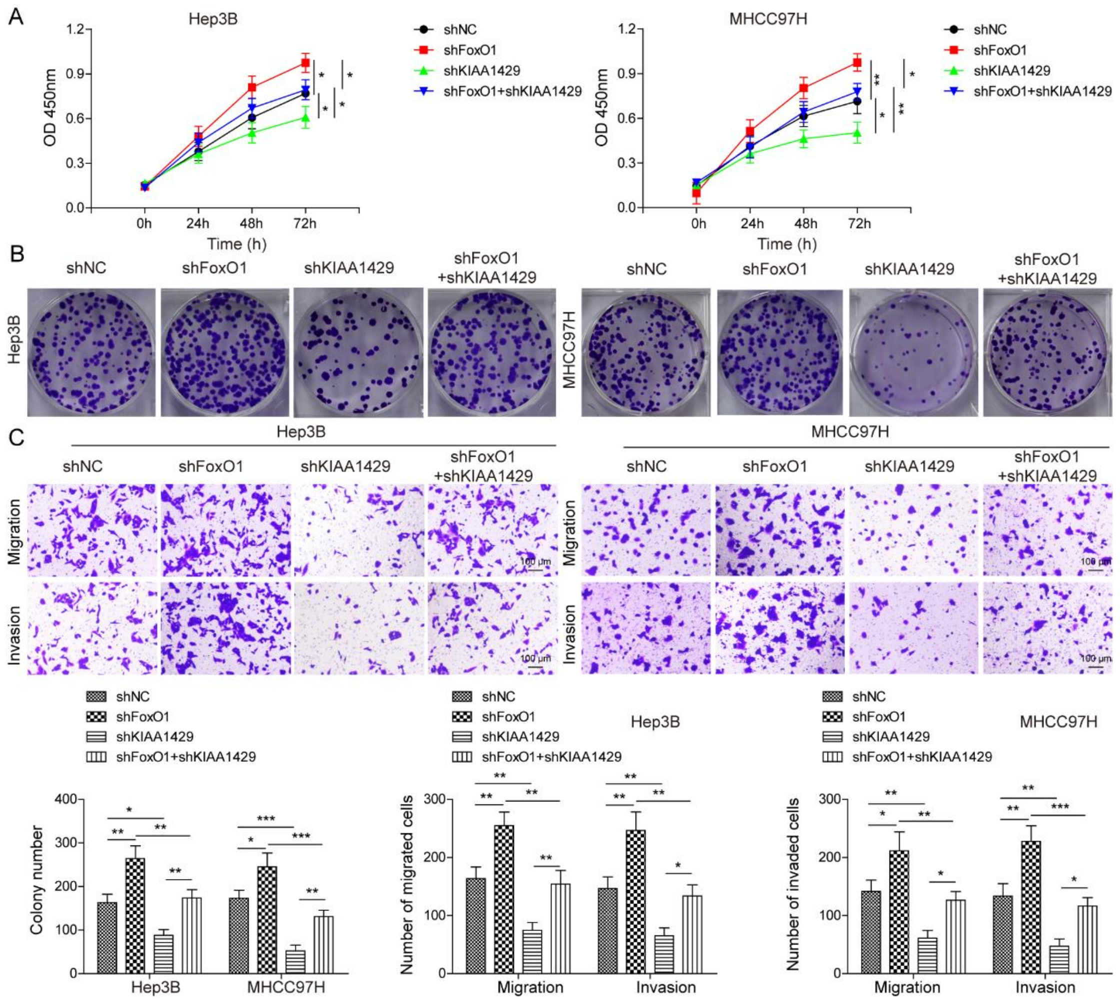

Supplementary Figure 6 The inhibitory effect of KIAA1429 silencing on HCC cell growth and metastasis was reversed by FoxO1 depletion. HCC cells were transfected with shFoxO1, shKIAA1429, or a combination of them. (A) CCK-8 and (B) colony formation assay detected the proliferative ability of HCC cells. (C) Transwell assay analysis of HCC cell migration and invasion (Scale bar = 100 μm). All experiments were repeated at least 3 times. *$p < 0.05$, **$p < 0.01$, ***$p < 0.001$.

2. Supplementary Table and Table legends

Supplementary Table 1 Oligonucleotide primer sets for qPCR.

Name	Sequence (5'¬3')	Length
KIAA1429 F	GAGTAAGAGCCCATAGCAGT	20

KIAA1429 R	TAGCACCAGACCATCAGTATTCAC	24
KDM5B F	TTCCACAGCTTGCTGAGATG	20
KDM5B R	GCCATAGCTTTCTCCACTGC	20
IFN-γ F	ACTGGCAAAAGGATGGTGAC	20
IFN-γ R	GACCTGTGGGTTGTTGACCT	20
Granzyme B F	AGACCCAGCAAGTCATCC	18
Granzyme B R	CAACCAGCCACATAGCAC	18
GAPDH F	ACAACTTTGGTATCGTGGAAGG	22
GAPDH R	GCCATCACGCCACAGTTTC	19

Supplementary Table 2 Correlation between KIAA1429 and clinicopathological characteristics in 60 patients.

Variable	KIAA1429		p value
	Low	High	
**Age (years)**			
≤50	13	19	0.1954
> 50	17	11	
**Sex**			
Female	12	17	0.3015
Male	18	13	
**HBsAg**			
Negative	9	12	0.5889
Positive	21	18	
**Liver cirrhosis**			
No	15	12	0.6042
Yes	15	18	
**Serum AFP, ng/mL**			
≤20	20	16	0.4296
> 20	10	14	
**Serum ALT, U/L**			
≤75	19	13	0.1954
> 75	11	17	
**Tumor size (diameter, cm)**			

≤5	22	9	0.0017**
> 5	8	21	
**Tumor number**			
Single	14	11	0.6010
Multiple	16	19	
**TNM**			
I/II	12	20	0.0692
III/IV	18	10	

**$P < 0.01$;
