# Supplementary figures and images for: Acetylated KIAA1429 by TIP60 facilitates metastasis and immune evasion of hepatocellular carcinoma via N6-methyladenosine-KDM5B-mediated regulation of FoxO1
Source: Cell Death Discov. 2025 Apr 29;11:210. doi: 10.1038/s41420-025-02462-4 (PMC12041376; doi:10.1038/s41420-025-02462-4)

Original Data
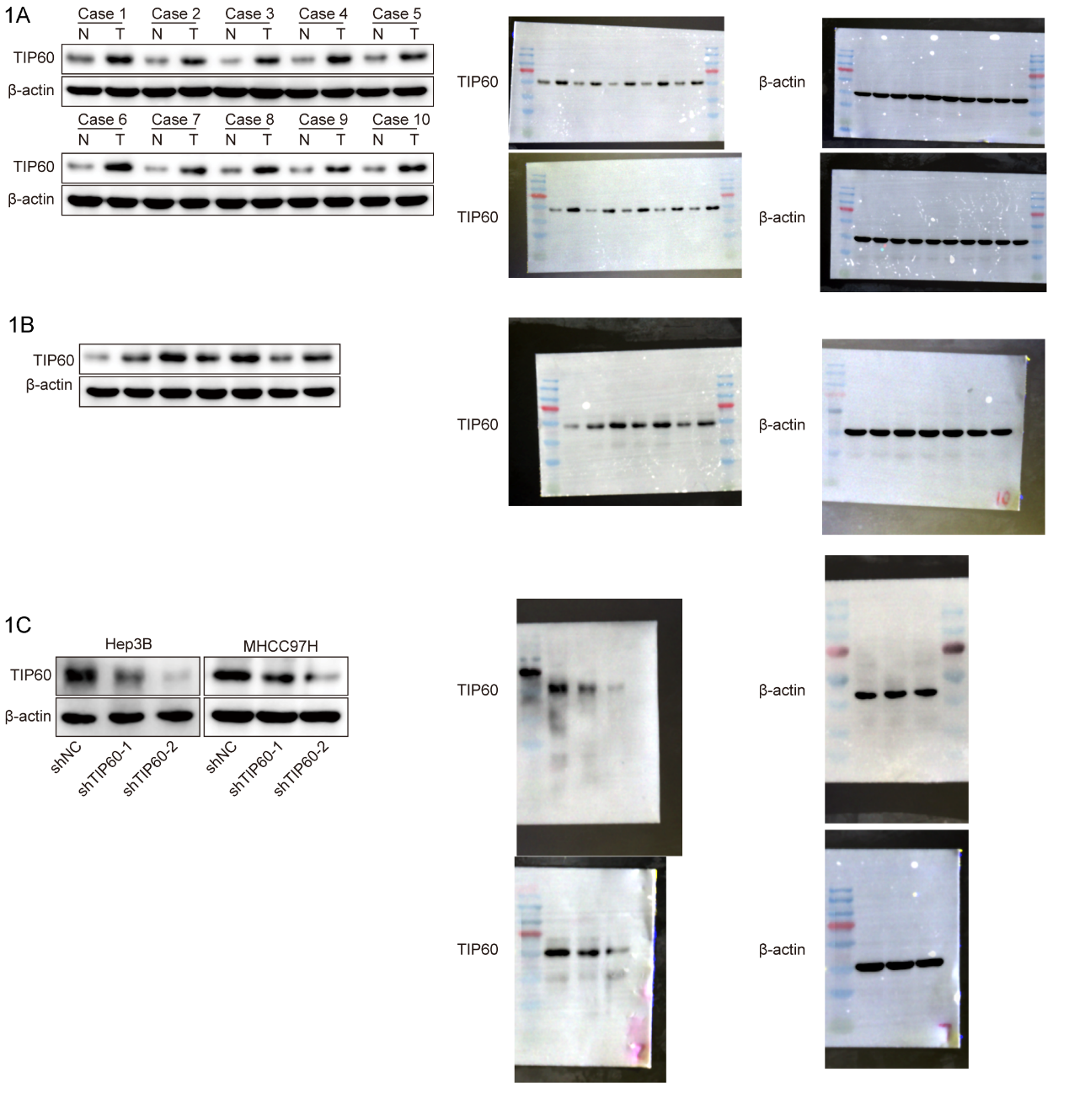


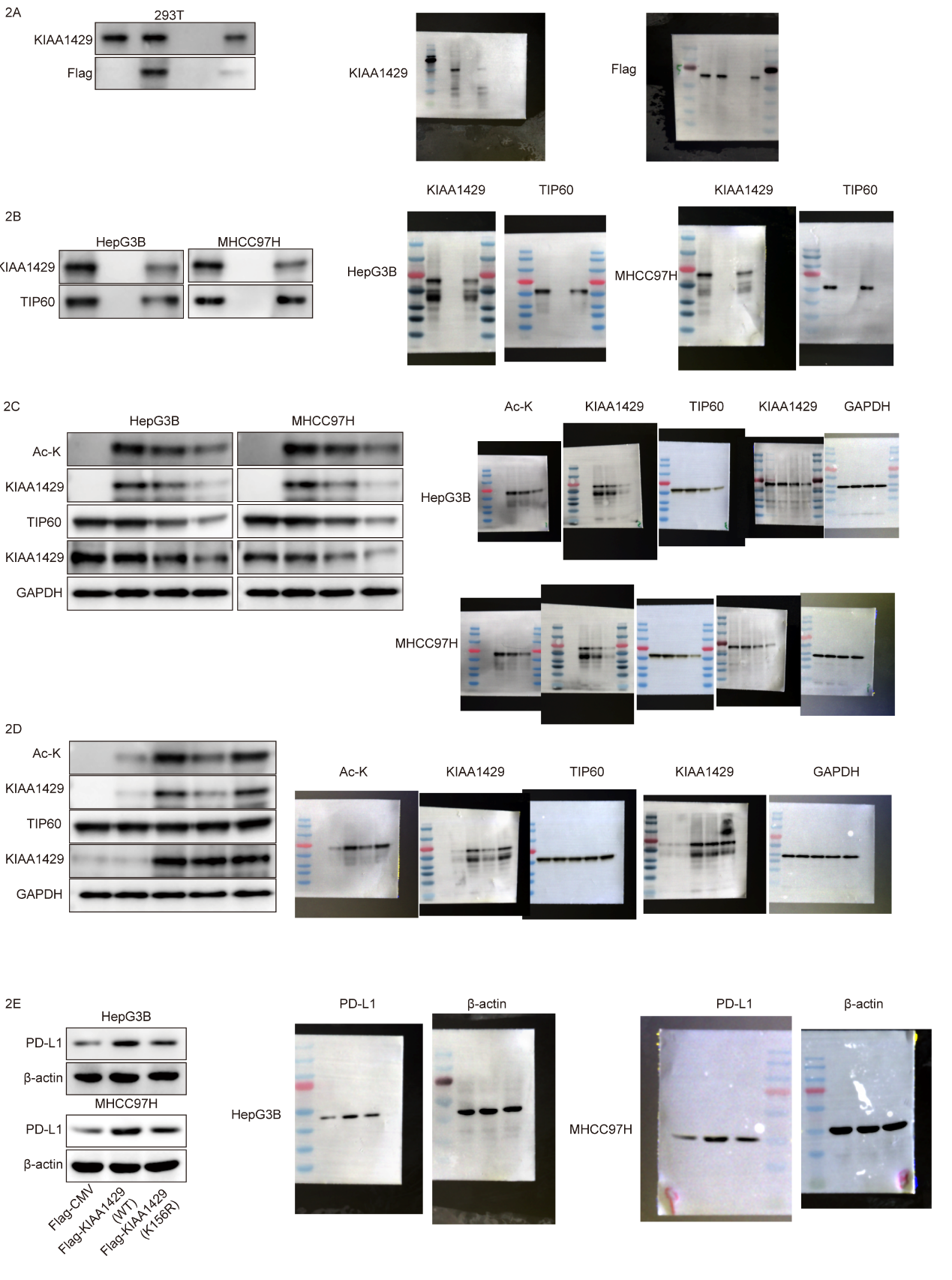


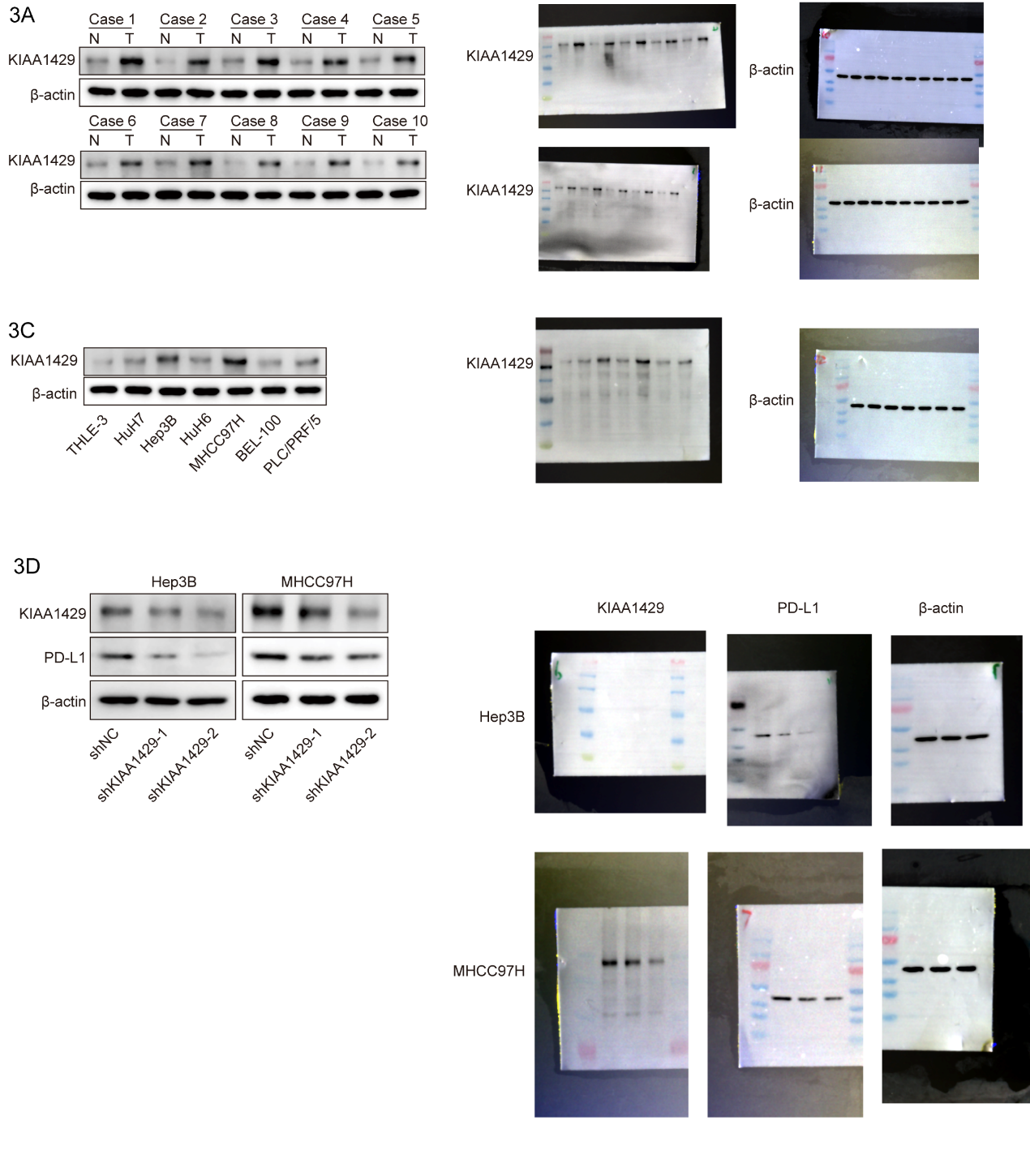


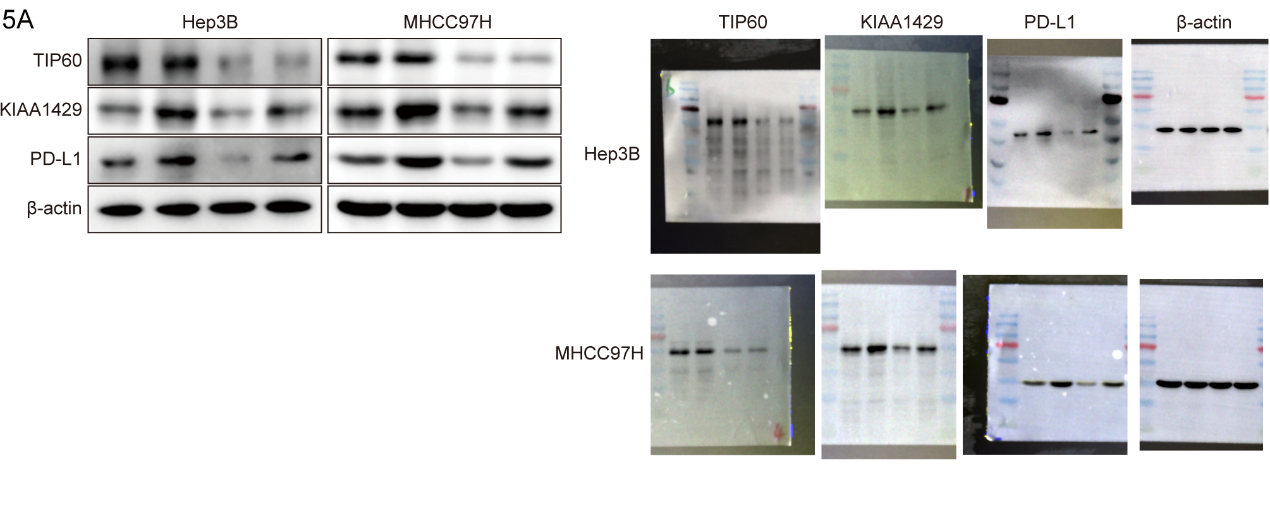


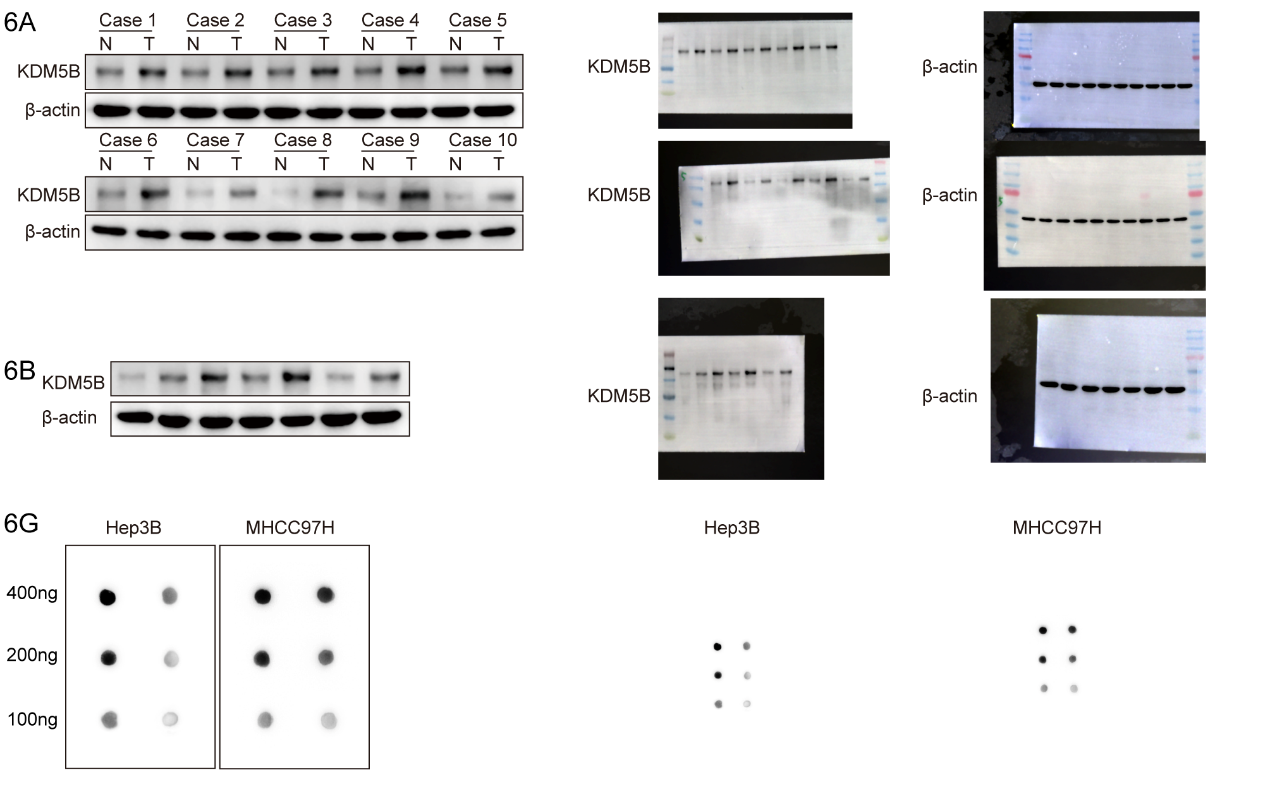


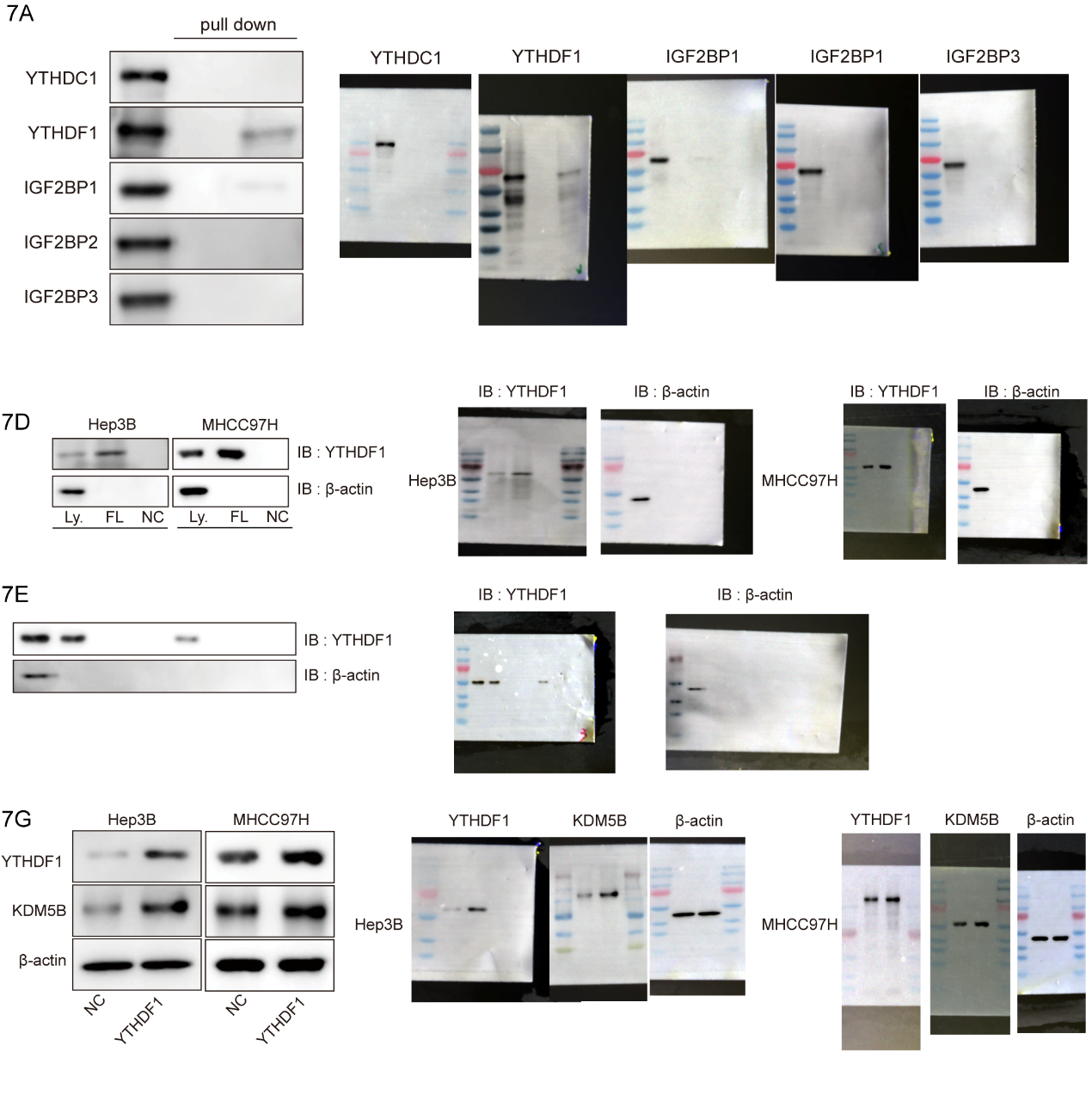


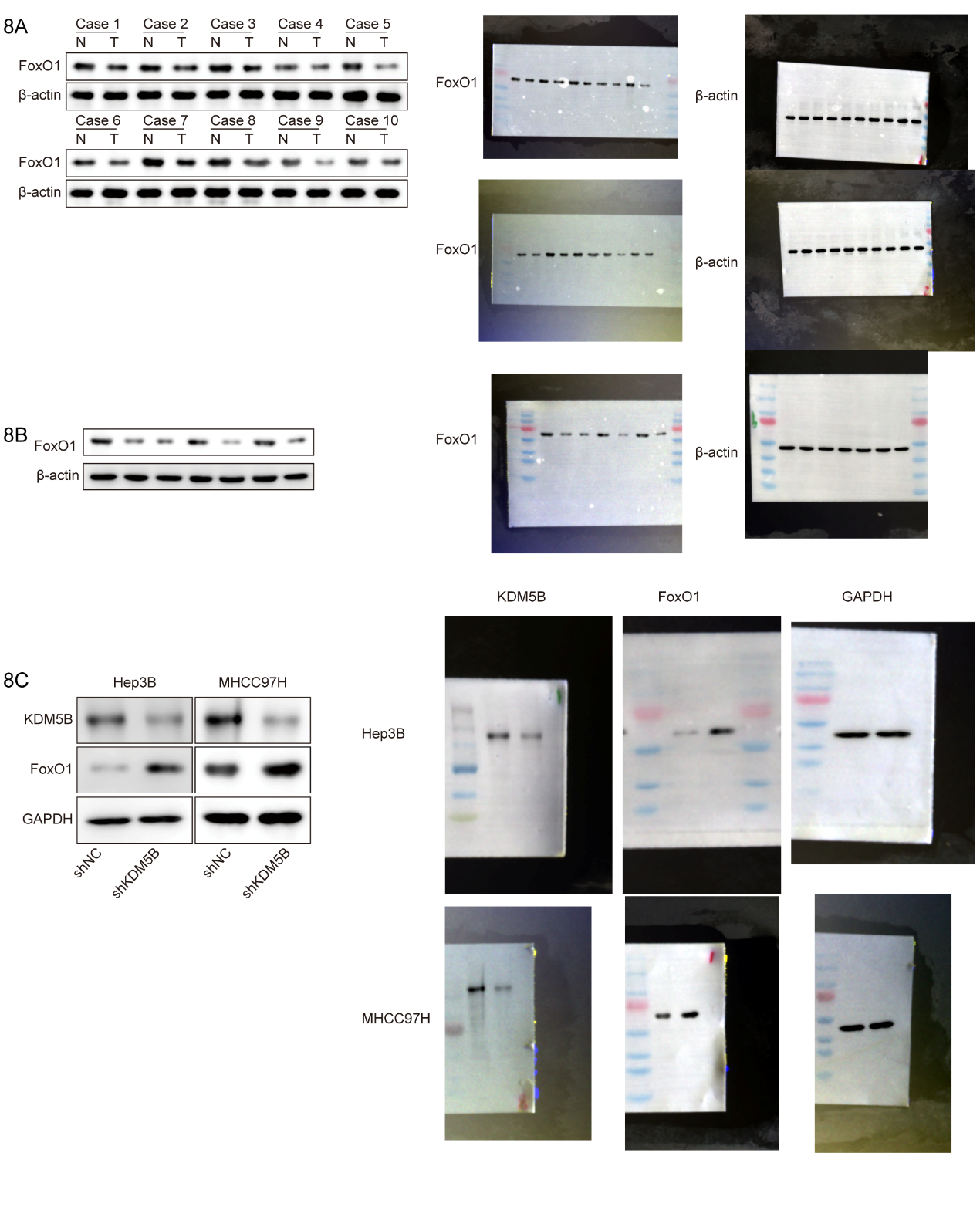


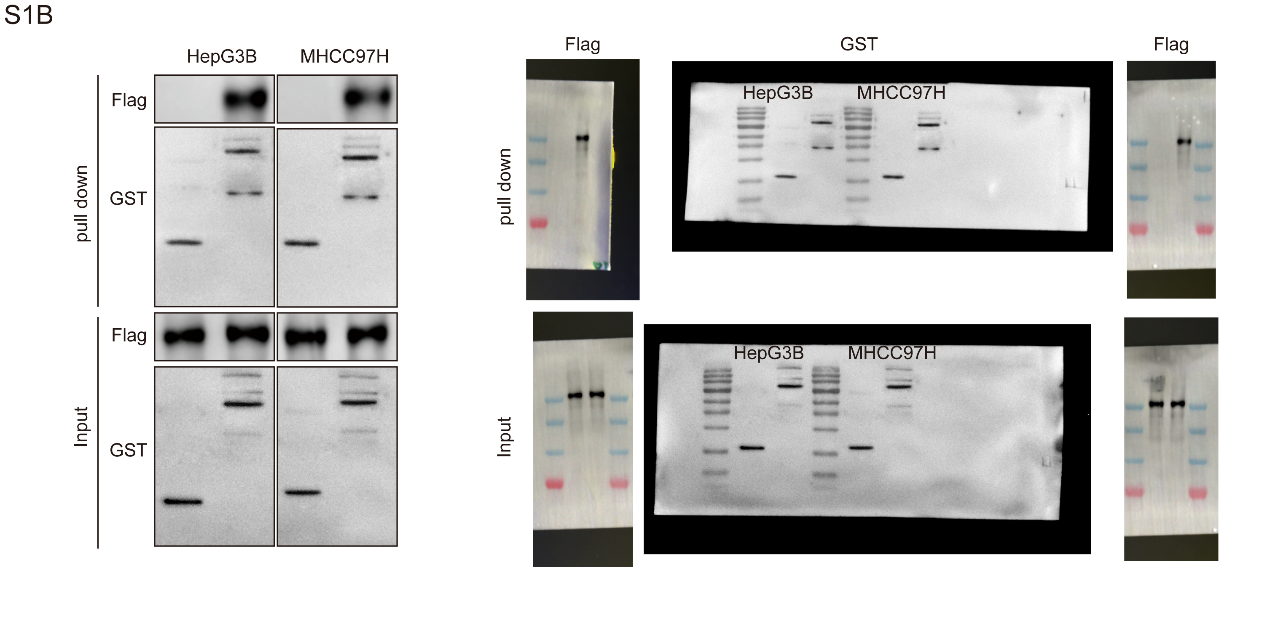


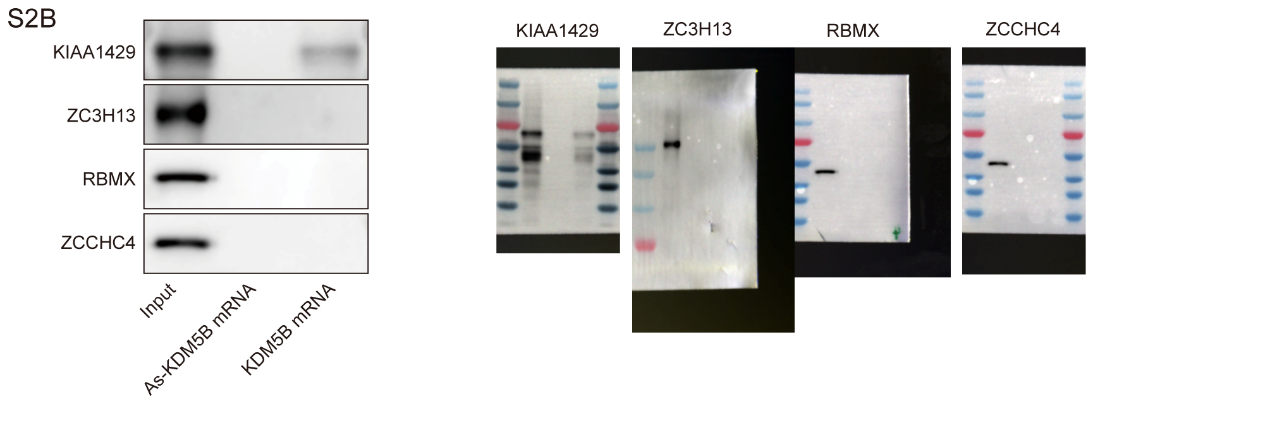


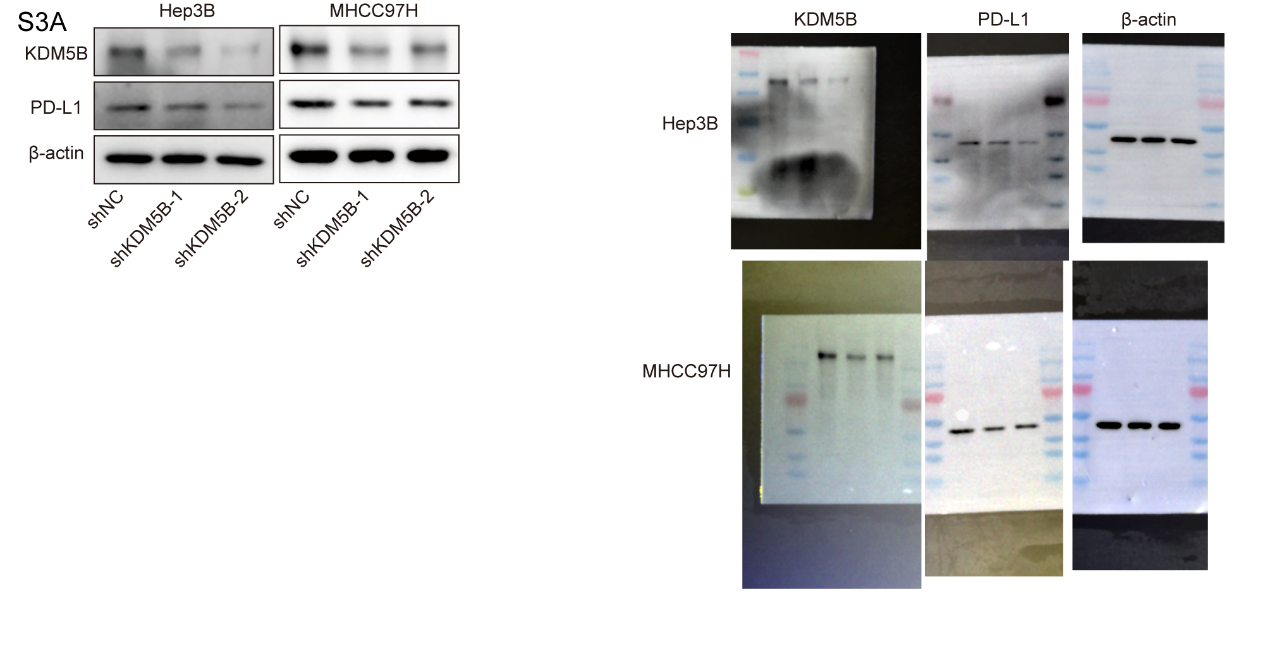


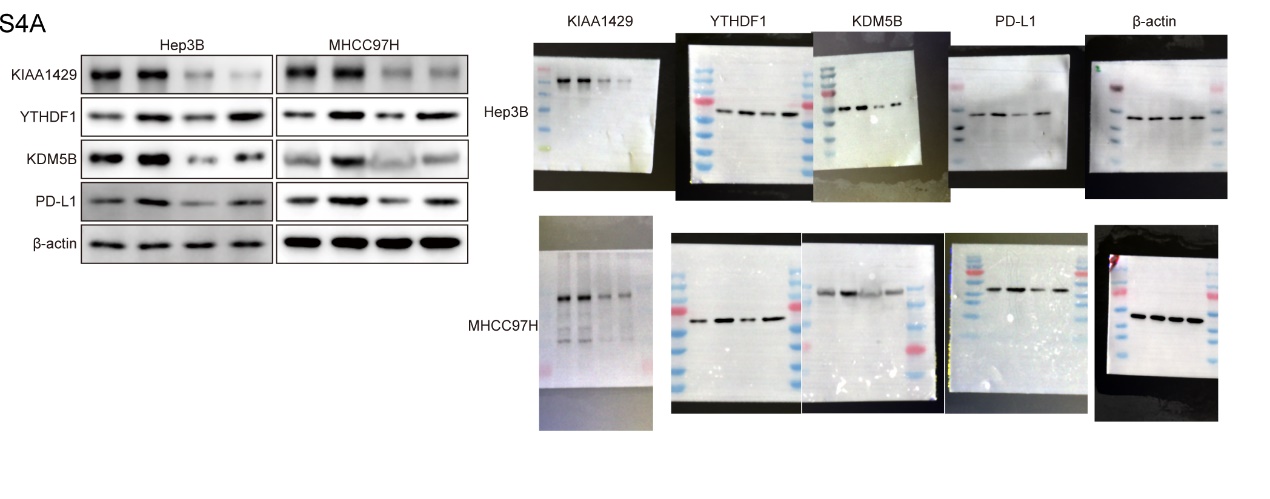


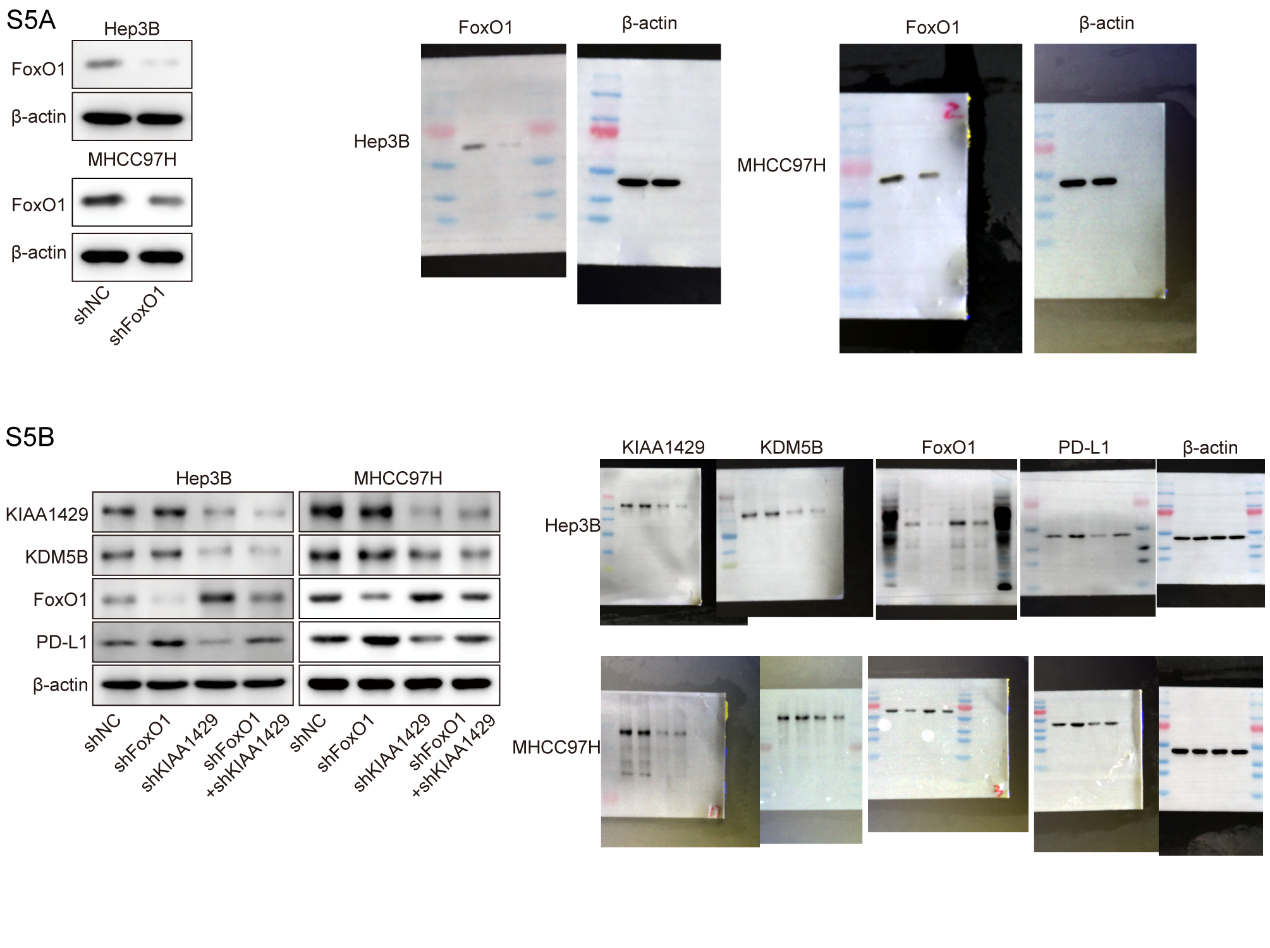

Supplement: Supplementary file 2 — Original data [file 41420_2025_2462_MOESM2_ESM.docx]
